# Water-detected NMR allows dynamic observations of repeat-expansion RNA condensates

**DOI:** 10.1038/s41557-025-01968-9

**Published:** 2025-10-15

**Authors:** Johannes Schmoll, Mihajlo Novakovic, Frédéric H-.T. Allain

**Affiliations:** Department of Biology, Institute of Biochemistry, ETH Zurich, Zurich, Switzerland

**Keywords:** RNA, Solution-state NMR, Biophysical chemistry

## Abstract

RNA condensation is implicated in the formation of neurotoxic RNA foci in cells affected by genomic expansions of trinucleotide or hexanucleotide repeats. However, the biophysical properties of repeat-expansion RNA condensates are poorly understood. Using CAG repeat-expansion RNA as a model system, we show that these RNA condensates cannot be observed with conventional nuclear magnetic resonance techniques. Therefore, we developed a nuclear magnetic resonance approach, based on water-detected semi-solid magnetization transfer, to detect and characterize RNA condensates in vitro. Our method, termed condensate detection by semi-solid magnetization transfer (CONDENSE-MT), is broadly applicable, highly sensitive and does not require direct observation of the biomolecules of interest. Using CONDENSE-MT, we could obtain dynamic information about RNA condensates, such as the relative amount and the tumbling rate of condensed RNA, the proton–solvent exchange kinetics and the amount of water molecules transiently bound in the condensate. We find that phase separation dramatically decreases molecular tumbling and is driven by heterotypic interactions between RNA and Mg^2+^. We further show that increasing CAG repeats decreases condensate hydration.

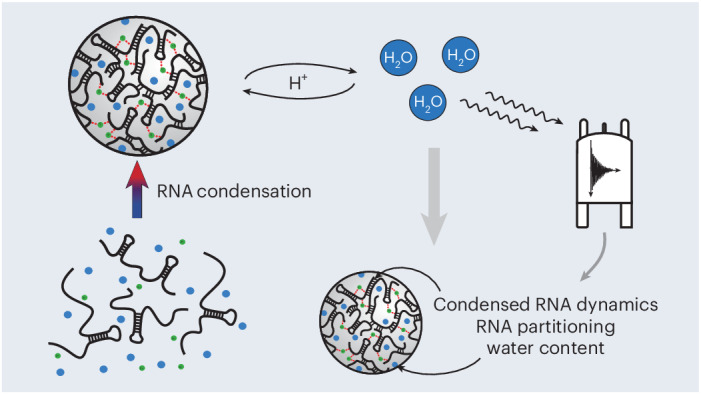

## Abstract (web summary)

The condensation of repeat-containing RNAs can have neurotoxic effects but is challenging to study. Now a NMR approach termed condensate detection by semi-solid magnetization transfer (CONDENSE-MT) can be used to study RNA condensate dynamics, proton–solvent exchange kinetics and condensate hydration.

## Main

Microsatellite repeat-expansion diseases are relentless neurodegenerative diseases, characterized by genomic expansions of trinucleotide or hexanucleotide repeat sequences. Prominent examples include expansions of CTG in myotonic dystrophy or CAG in Huntington’s disease^1–3^. Disease onset is strictly correlated with a disease-specific threshold number of repeats, yet the molecular mechanism underlying disease progression is poorly understood^4–7^. A hallmark of repeat-expansion diseases is the formation of microscopically visible nuclear foci^3,8–11^, composed of transcribed repeat-expansion RNA, a class of RNA that has recently been shown to form droplet-shaped condensates in vitro in the absence of proteins^3,12^. While early studies assumed that the ability to form droplets was unique to disease-associated repeat-expansion RNA^3,13^, emerging evidence suggests that RNA polymers are generally capable of condensation, depending on solution conditions^12,14^. The reason why repeat-expansion RNAs form visible nuclear foci, while other RNAs do not, remains unknown. However, similar to disease onset, the formation of RNA foci relies on repeat-expansion RNA exceeding a threshold number of repeats, suggesting that condensates formed by disease-associated repeat-expansion RNAs possess unique biophysical properties that allow foci formation in vivo^3,5,10^. RNA condensation in vitro usually occurs through a lower critical solution temperature (LCST)-type phase transition, where droplet formation is triggered at temperatures above a defined threshold^12,14^. This contrasts with the traditional enthalpy-driven framework of liquid–liquid phase separation commonly used to describe the formation of membraneless organelles in eukaryotic cells^15–18^. However, the necessity for elevated temperatures to induce RNA condensation in vitro limits the accessibility of this phenomenon to conventional experimental approaches. Furthermore, currently available methodology for characterization of biological condensates commonly involves labelling the biomolecule of interest with fluorescent labels, a strategy that greatly enhanced our understanding of biomolecular phase separation, but has also been shown to alter biomolecular structure, localization or phase separation behaviour^19–22^. As a consequence, the biophysical properties of these condensates remain poorly understood, and there is an urgent need for label-free methods to probe disease-associated RNA condensates. Using CAG repeat-expansion RNA as a model system, we developed a non-invasive, label-free method, based on nuclear magnetic resonance (NMR) spectroscopy, to study RNA condensates in vitro. Our approach, termed condensate detection by semi-solid magnetization transfer (CONDENSE-MT) is based on water-detected NMR off-resonance semi-solid magnetization transfer (MT), a phenomenon previously exploited for generating contrast during magnetic resonance imaging^23–25^. For method development, we focused on RNA oligomers consisting of 31 repeats of CAG, (5′-CAG-3′)_31_ commonly referred to as 31xCAG, since this length lies at the threshold of disease onset for several CAG repeat-expansion disorders^26^ but is still amenable for efficient in vitro synthesis. Using CONDENSE-MT, we could precisely quantify the relative population of condensed protons under biphasic conditions, which reports on the overall amount of condensate-associated (5′-CAG-3′)_31_. Furthermore, we could extract proton exchange kinetics between bulk H_2_O and the RNA condensate, the *T*_2_ relaxation constant of nuclei associated with condensed RNA, which provides information on its tumbling rate, and the *R*_2_ relaxation rate of bulk H_2_O in the presence of condensates, which reports on slow-tumbling water molecules associated with the condensate. The study presented here introduces a broadly applicable method for the characterization of NMR-invisible condensates, which requires only single-scan solvent detection and provides important insights into the biophysical properties of repeat-expansion RNA condensates in vitro.

## Results

### Reconstitution of (5′-CAG-3′)_31_ condensates

**Fig. 1:**
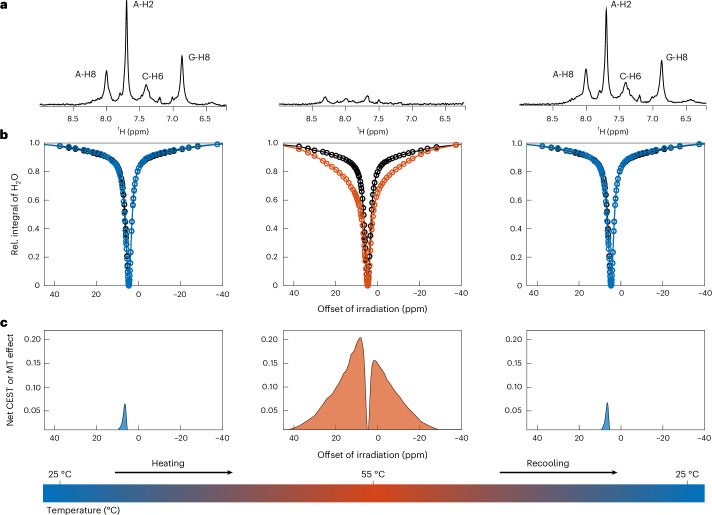
Indirect observation of (5′-CAG-3′)_31_ condensates by solvent detection. **a**, 1D ^1^H-NMR spectrum of the aromatic and amino region of (5′-CAG-3′)_31_ (150 μM) at 25 °C (left panel), at 55 °C (central panel) and after recooling to 25 °C (right panel) in presence of an agarose meshwork (0.5%, w/v) and 50 mM MgCl_2_. Assignments indicate aromatic ^1^H resonances of adenine (A), guanine (G) and cytosine (C) and are given within the spectrum. **b**, Normalized integral of bulk H_2_O as a function of offset saturation in presence of (5′-CAG-3′)_31_ at 25 °C (left panel), at 55 °C (central panel) and after recooling to 25 °C (right panel), shown in blue (25 °C) or orange (55 °C). For comparison, the reference profile of the agarose meshwork was recorded at 25 °C (left and right panels) and at 55 °C (central panel), shown in black. Offset saturation occurred with a B_1_ amplitude of 250 Hz. **c**, Net effect of (5′-CAG-3′)_31_ on the offset saturation of solvent water, determined via subtraction of the relative water integrals shown in **b**. **a**–**c**, All experiments were performed at 700-MHz *B*_0_ field strength. Source data

**Extended Data Fig. 1:**
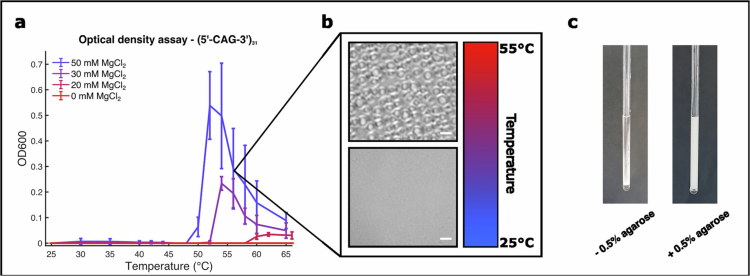
Establishment of (5′-CAG-3′)_31_ condensates as a model system. **a** Optical density at 600 nm of (5′-CAG-3′)_31_ (40μM) as a function of temperature and MgCl_2_ concentration. All experiments were performed in three independently prepared and measured replicates. Error bars indicate standard deviation from the average of the three replicates. **b** Light microscopy of (5′-CAG-3′)_31_ (37.5μM) in presence of 50 mM MgCl_2_, at 25 °C and after heating to 55 °C. Scalebar indicates 2 μm. **c** Incubation of biphasic (5′-CAG-3′)_31_ (150 μM, at 50 mM MgCl_2_) in 3 mm NMR tubes in a temperature-controlled water bath for 2.5 h at 55 °C in absence (*left*) and in presence (*right*) of a 0.5% (w/v) agarose meshwork. Source data

**Extended Data Fig. 2:**
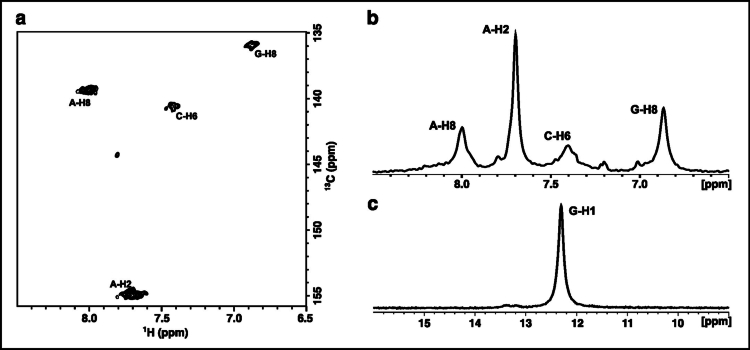
Spectral analysis of (5′-CAG-3′)_31_. **a** ^1^H,^13^C HMQC spectrum of aromatic resonances of (5′-CAG-3′)_31_ at 25 °C. Assignment is given for each resonance and indicates aromatic proton resonances for adenine (A), guanine (G) and cytosine (C). The spectrum was recorded using natural abundance of ^13^C at 25 °C using 150 μM (5′-CAG-3′)_31_ in presence of 10 mM MgCl_2_ in 25 mM Tris/HCl, pH 7.4. **b** 1D ^1^H NMR spectrum of the aromatic region (6.5–8.5 ppm) recorded under water suppression. Assignments are given according to the HMQC spectrum shown in **a**. **c** SOFAST ^1^H spectrum of the (5′-CAG-3′)_31_ imino-region (9 to 16 ppm) to visualize GC base-pairing patterns of the (5′-CAG-3′)_31_ oligomer. **b** and **c** Spectra were recorded at 25 °C using 150 μM (5′-CAG-3′)_31_ in presence of 50 mM MgCl_2_ in 25 mM Tris/HCl, pH 7.4.

**Extended Data Fig. 3:**
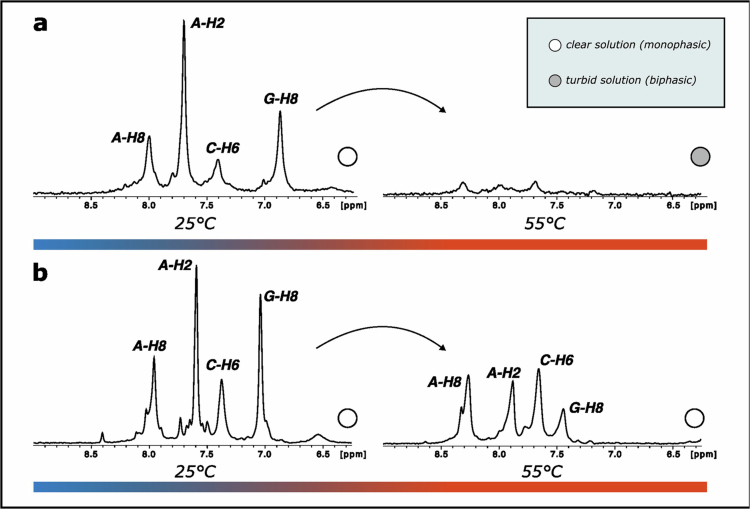
1D NMR spectroscopy of RNA condensation. **a** 1D ^1^H-NMR spectra (aromatic/amino region) of (5′-CAG-3′)_31_ (150 μM, at 50 mM MgCl_2_) at 25 °C (monophasic) and at 55 °C (biphasic), prepared in 0.5% (w/v) agarose. **b** Control 1D ^1^H-NMR spectra of (5′-CAG-3′)_31_ (150 μM) in 0.5% (w/v) agarose at 25 °C and 55 °C in absence of MgCl_2_. **a** and **b:** All samples were prepared in 25 mM Tris/HCl, pH 7.4 and 5% D_2_O. Conditions where (5′-CAG-3′)_31_ condenses into turbid, biphasic mixtures are indicated by white and grey spheres for each NMR spectrum.

Condensation of RNA in vitro occurs by a LCST-type phase transition with MgCl_2_ concentration playing a crucial role. At low temperatures, phase separation is suppressed, resulting in monophasic mixtures, whereas heating above a threshold temperature induces condensation, leading to biphasic systems^12,14^. Phase separation of RNA has been reported to occur around 40–60 °C (refs. ^12,14^). To develop a robust model system for investigating RNA condensates, we determined the LCST of a (5′-CAG-3′)_31_ oligomer to be 52 °C at 50 mM MgCl_2_, consistent with previous reports^12^. Notably, the LCST increased at decreasing MgCl_2_ concentrations (Extended Data Fig. 1a). Imaging confirmed spherical, droplet-like condensates of (5′-CAG-3′)_31_ above the LCST (Extended Data Fig. 1b). To stabilize biphasic samples and prevent sedimentation of the dense phase, we embedded the condensates in a 0.5% agarose meshwork, a technique commonly used for NMR spectroscopy of multiphasic samples^27,28^. The absence of sedimentation of the dense phase over time demonstrates the capability of agarose to stabilize multiphasic samples even at elevated temperatures (Extended Data Fig. 1c). (5′-CAG-3′)_31_ is engaged in a highly regular folding pattern under monophasic conditions, leading to single, well-defined resonances for A_H2_, A_H8_, C_H6_ and G_H8_ in ^1^H-^13^C heteronuclear single quantum coherence spectra (Extended Data Fig. 2a,b) and a single ^1^H resonance in the imino-region, reporting on conventional Watson–Crick GC base pairing (Extended Data Fig. 2c)^29^. We conclude that most RNA bases are engaged in structurally similar double-strand interactions, which precludes sequence-specific resonance assignment or residue-specific information about (5′-CAG-3′)_31_ via NMR spectroscopy. Condensation of (5′-CAG-3′)_31_ resulted in a substantial loss of observable ^1^H resonances for the oligomer (Fig. 1a). While we can attribute the loss of resonances corresponding to labile protons due to an increase in chemical exchange with bulk H_2_O at higher temperatures, this cannot account for the strong reduction of resonances corresponding to aromatic protons (Fig. 1a and Extended Data Fig. 2a). Furthermore, monophasic control samples lacking MgCl_2_ retained observable ^1^H resonances of (5′-CAG-3′)_31_ on temperature shift (Extended Data Fig. 3a,b). This suggests that, on phase separation, most of (5′-CAG-3′)_31_ is sequestered into a dense phase that is not observable by conventional NMR techniques, while a minor fraction of RNA remains in the dilute phase, contributing to the weak signals observed under biphasic conditions. Recooling to 25 °C restored the initial, monophasic signal, indicating that the condensation process is largely reversible (Fig. 1a).

### Indirect detection of (5′-CAG-3′)_31_ condensates

**Extended Data Fig. 4:**
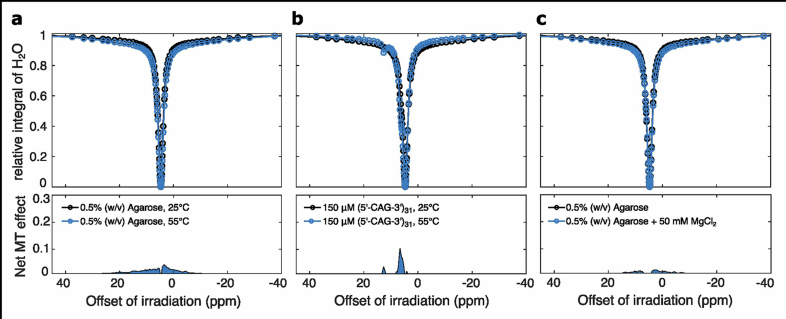
Control experiments for water-detected magnetization transfer effects. **a** Relative water integral as function of offset saturation at 170 Hz B_1_ amplitude (MT profile) of an agarose meshwork (0.5%, w/v) at 25 °C and 55 °C (*upper panel*) in the absence of (5′-CAG-3′)_31_. Difference of the MT profiles (Net MT-effect of the temperature shift on agarose) is shown below. **b** MT profile at 170 Hz B_1_ amplitude of (5′-CAG-3′)_31_ in absence of MgCl_2_ but in presence of an agarose meshwork (0.5%, w/v). Difference between the MT profiles (Net MT-effect of (5′-CAG-3′)_31_ in absence of MgCl_2_) is shown below. **c** MT profile at 170 Hz B_1_ amplitude of the agarose meshwork (0.5%, w/v) at 55 °C in absence and presence of 50 mM MgCl_2_. Difference of the MT-profiles (net effect of MgCl_2_ on agarose) is shown below. Source data

The absence of detectable ^1^H resonances for condensed (5′-CAG-3′)_31_ indicates the formation of large, dynamically arrested assemblies on droplet formation. The resulting, substantial reduction in molecular tumbling decreases RNA *T*_2_ constants and thus broadens condensate-associated NMR resonances beyond detection by conventional NMR methods. Furthermore, indirect methods, such as the dark-state exchange saturation transfer approach, are inapplicable to our system due to an insufficient dilute-phase RNA signal (Fig. 1a) and the absence of chemical exchange of RNA between the two phases^3,12,30,31^. We proposed that, instead of physical exchange of RNA molecules, simple proton exchange could relay the relaxation properties of the NMR-invisible condensates to observable species in solution. In aqueous environments, proton exchange inevitably involves solvent H_2_O, allowing the relaxation properties of condensed RNA to be directly encoded in bulk water. Given the high concentration of H_2_O in biochemical solutions, a water-detected approach also offers substantial sensitivity enhancements, commonly exploited in chemical exchange saturation transfer (CEST) experiments to detect low-concentration solutes^25,32,33^. To exploit this effect, we irradiated our biphasic (5′-CAG-3′)_31_ mixture with a weak transverse magnetic field (*B*_1_) across a range of offset frequencies to saturate ^1^H resonances at the respective offsets and allowed for chemical exchange of the saturated nuclei with bulk water. Here, we defined the frequency offset of saturation according to standard nomenclature for proton NMR spectra where the water ^1^H resonance appears at ~4.7 ppm with the zero-frequency set at the ^1^H resonance of tetramethylsilane. We then measured the attenuation of solvent H_2_O as a function of offset irradiation frequency to extract solvent relaxation properties. Comparative analysis of water relaxation as a function of biomolecular condensation then provided insights into the relaxation properties of (5′-CAG-3′)_31_ condensates, relayed to water by proton exchange. This revealed a striking attenuation of the H_2_O resonance in the presence of (5′-CAG-3′)_31_ condensates within an offset saturation range of ±40 kHz at a *B*_1_ field strength of 250 Hz: a line width that is consistent with semi-solid structures (Fig. 1b,c)^24,34^. Detection of this semi-solid species required temperature-induced condensation of (5′-CAG-3′)_31_ as this effect was absent at 25 °C (Fig. 1b,c) and required the presence of both (5′-CAG-3′)_31_ and MgCl_2_ (Extended Data Fig. 4a,b), which agrees with optical density assays and direct NMR spectroscopy of (5′-CAG-3′)_31_ (Extended Data Figs. 1a and 3b). Furthermore, we could not detect any MgCl_2_-dependent effects in absence of RNA (Extended Data Fig. 4c). This effect was reversible on cooling the sample back to 25 °C, consistent with the recovery of observable RNA resonances (Fig. 1a–c). For all monophasic conditions, we instead observed a liquid-state CEST pool at a chemical shift between 5 ppm and 12 ppm, consistent with an averaged CEST effect of RNA imino (12 ppm) and amino (6 ppm) resonances (Fig. 1c and Extended Data Fig. 4b).

Given the semi-solid nature of reconstituted (5′-CAG-3′)_31_ condensates, classical CEST formalisms are inadequate for quantitative description, as the underlying Bloch–McConnell equations would indicate a Lorentzian line shape characteristic of liquid-state CEST pools^34–36^. Instead, we next used quantitative, semi-solid MT formalisms, as commonly used in magnetic resonance imaging to exploit chemical exchange from immobile, tissue-associated protons to mobile H_2_O for tissue contrast (MT contrast)^23,24,36^, and adapted them to our system.

### Quantitative framework for RNA condensate characterization

**Fig. 2:**
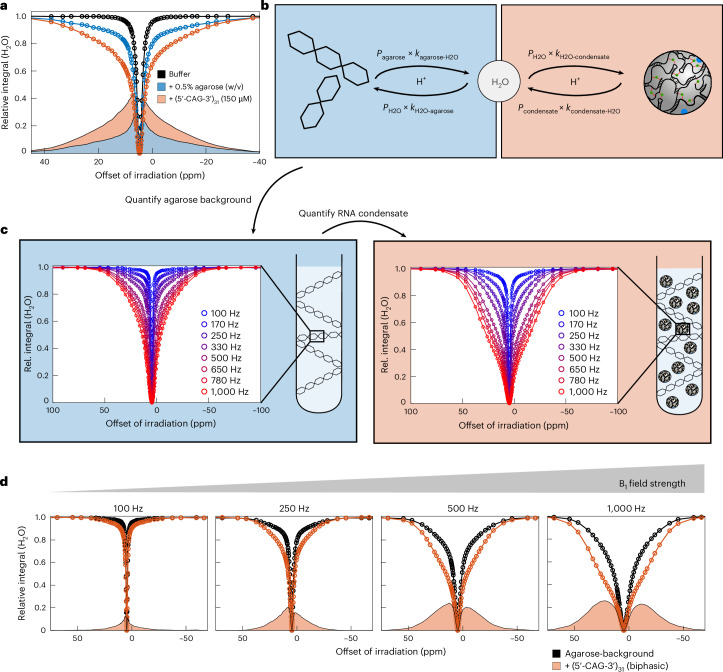
RNA condensate characterization via multi-step multi-parameter global fitting of MT data (CONDENSE-MT). **a**, Comparison of water-detected MT profiles of aqueous buffer, in presence of 0.5% (w/v) agarose and in presence of (5′-CAG-3′)_31_ condensates embedded in agarose. MT profiles were recorded at 55 °C in presence of 50 mM MgCl_2_. **b**, Schematic representation of the equilibrium chemical exchange model used between the agarose meshwork, the semi-solid RNA condensate and solvent H_2_O. *P*, population; *k*, exchange rate constant. **c**, Water-detected MT profiles of the agarose meshwork (0.5% w/v, left, indicated in blue) and of (5′-CAG-3′)_31_ condensates (150 μM) embedded in agarose (right, indicated in orange) at different *B*_1_ field strengths (given in Hertz for each profile). **d**, MT effect of the agarose meshwork in absence of (5′-CAG-3′)_31_ (black) and biphasic (5′-CAG-3′)_31_, reconstituted in agarose (orange), for different *B*_1_ field strengths given above in Hertz for each panel. Net effect of (5′-CAG-3′)_31_ (obtained via subtraction) is shown as orange areas. Source data

**Extended Data Fig. 5:**
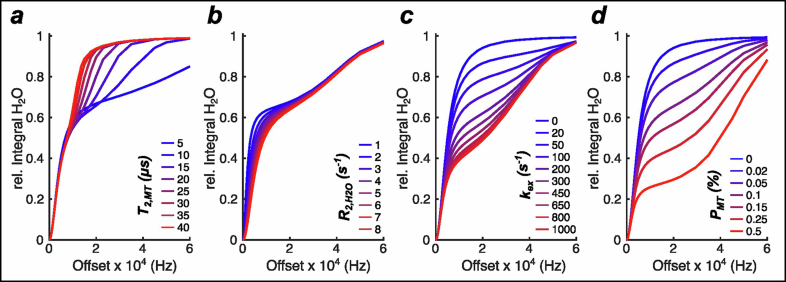
CONDENSE-MT simulations. Simulation of the relative integral of bulk water as function of offset saturation (given in Hz, relative to the water reference) in presence of a semi-solid MT pool (at Gaussian line shape), by systematically varying T_2,condensate_ (**a**), ${R}_{2,{\mathrm{H}}_2{\rm{O}}}$ (**b**), k_ex_ (= k_MT,water_ + k_water,MT_) (**c**) or P_MT_ (**d**). Unless systematically varied as indicated, parameters for simulation were: T_2,condensate_ = 8μs, ${R}_{2,{\mathrm{H}}_2{\rm{O}}}$ = 5 s^−1^, k_MT,water_ = 150 s^−1^, k_water,MT_ = 0.15, P_condensate_ = 0.1%, B_1_ = 1000 Hz, R_1,water_ = 0.2 s^−1^.

For quantitative characterization of RNA condensates, we derived a model that accounts for two independent semi-solid MT pools: the agarose meshwork and the RNA condensate (Fig. 2a). Since semi-solid MT relies on proton exchange from immobile, semi-solid material to bulk H_2_O, our model captures the equilibrium chemical exchange between two MT pools and solvent H_2_O (Fig. 2b). We implemented this model within the framework of MT contrast theory^23,34,36^, while expanding it to accommodate two independent MT pools (Methods). Bulk H_2_O engages in simultaneous chemical exchange with both semi-solid species. We therefore first determined the physical parameters of the agarose meshwork in isolation and held these parameters constant during subsequent quantification of RNA condensates embedded in an agarose meshwork of equal concentration (Fig. 2c). This stepwise approach assumes independence of the two MT pools, which agrees with our three-site exchange model where we explicitly excluded direct proton exchange between agarose and the RNA condensate (Fig. 2b). Given the low concentrations of agarose (0.5% w/v) and RNA (150 µM) relative to bulk H_2_O, this assumption is reasonable. Increasing the B_1_ field strength during offset saturation extended the apparent line width of RNA condensates (Fig. 2c,d), indicating that physical parameters of the condensate might not reliably be extracted from a single experiment using one nutation frequency for offset saturation. This agrees with earlier MT contrast studies, showing that offset saturation over a range of *B*_1_ field strengths is required for full description of a semi-solid system^24^. Notably, the presence of (5′-CAG-3′)_31_ condensates at an RNA concentration of 150 µM led to a maximal water attenuation of up to 25%, resulting in a 150-fold signal amplification by ensuing water detection (Fig. 2d and Supplementary Note 1). According to this experimental design, we named our approach CONDENSE-MT. CONDENSE-MT simulations demonstrate that our approach can distinguish molecular species with *T*_2_ constants below 30 μs (Extended Data Fig. 5a). Furthermore, our simulations revealed a spectral region where our model is exclusively sensitive to the *R*_2_ rate of bulk water, which is commonly elevated in presence of semi-solids due to motion-restricted water molecules directly associated with the MT pool (‘bound water’)^24,37–39^ (Extended Data Fig. 5b). Thus, besides extracting the physical properties of condensed RNA, CONDENSE-MT can detect slow-tumbling water in the condensates. Finally, using offset saturation at *B*_1_ field strengths up to 1,000 Hz (Fig. 2d) enables sensitive quantification of overall proton exchange rates up to 600 s^−1^ and low RNA populations (Extended Data Fig. 5c,d).

### Quantification of (5′-CAG-3′)_31_ condensates via CONDENSE-MT

**Fig. 3:**
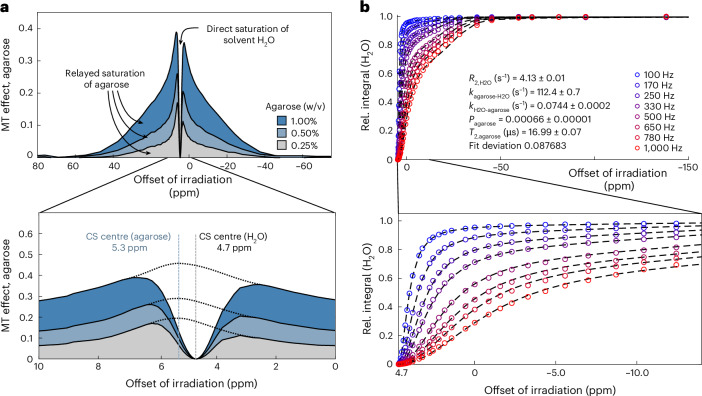
Characterization of the agarose meshwork. **a**, Net MT effect of agarose (compared with aqueous buffer) at different concentrations of agarose at 250 Hz offset saturation. Chemical shift centre of semi-solid agarose was determined by interpolation, assuming symmetry of the agarose resonance, and is indicated in the panel below. Areas corresponding to direct self-saturation of water and to relayed saturation from agarose are indicated. CS, chemical shift. **b**, Multi-parameter global fitting of the water-detected MT profile of 0.5% (w/v) agarose at 55 °C. Experimentally recorded MT profile is shown as a scatter plot; theoretical prediction after computational optimization is given as dashed line. The MT profile at spectral region close to the H_2_O resonance (4.7 ppm) is shown enlarged below. Saturation amplitudes (*B*_1_) are given in Hertz. Parameter errors were estimated from the diagonal elements of the variance–covariance matrix resulting from fitting experimental data to the CONDENSE-MT model. Fit deviation indicates overall deviation of experimental datapoints from the model prediction after computational optimization. Source data

We next sought to quantify the agarose meshwork in the absence of RNA via global multi-parameter fitting to our model (Fig. 2b,c). We estimated a centre of the semi-solid agarose MT effect of approximately 5.3 ppm via interpolation, consistent with the carbohydrate hydroxyl-protons as the primary source of proton exchange (Fig. 3a). For fitting, we generally restricted our analyses to saturation offsets upfield of the H_2_O resonance (−150 ppm to 4.7 ppm) to omit effects from liquid-state CEST pools, given that any liquid-state CEST effects are expected at a spectral range between 5 ppm and 15 ppm for exchangeable NH or OH protons in biomolecules (Fig. 1c and Extended Data Fig. 4b). Fitting agarose data to our model yielded excellent agreement with experimental data (Fig. 3b). Therefore, we fixed the physical parameters of the agarose meshwork in our model for subsequent detection and quantification of (5′-CAG-3′)_31_ condensates. For a more elaborate discussion of the agarose meshwork parameters, we refer to Supplementary Note 2.

**Fig. 4:**
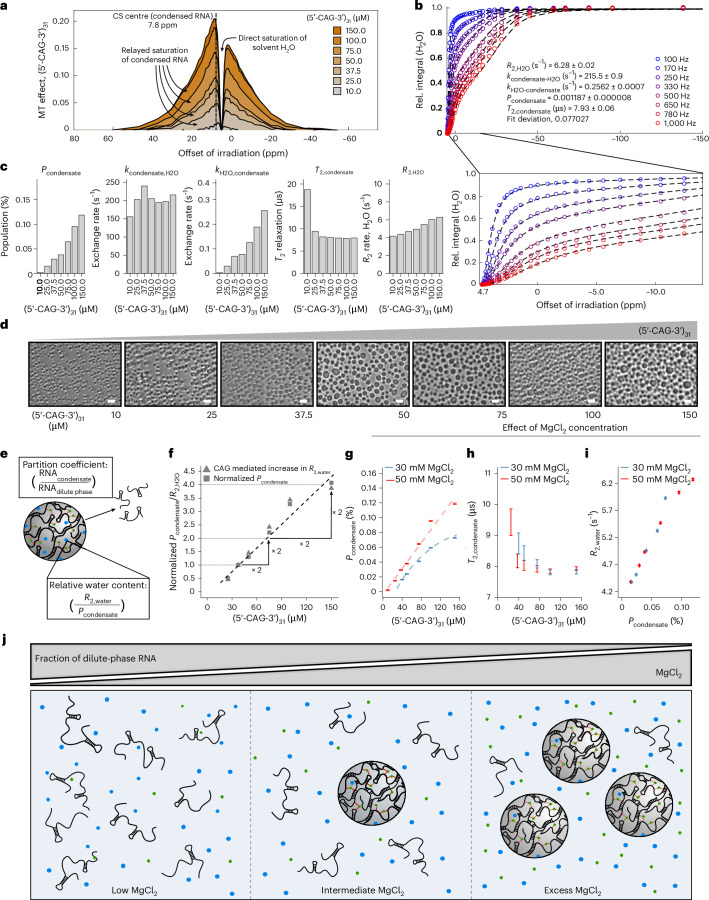
Characterization of RNA condensates as a function of overall RNA concentration. **a**, Net MT effect of biphasic (5′-CAG-3′)_31_, compared with the agarose meshwork (0.5%, w/v), at different concentrations of overall RNA at 250-Hz offset saturation. **b**, Quantification of (5′-CAG-3′)_31_ (150 μM), embedded in agarose (0.5% w/v) via CONDENSE-MT. Physical parameters of agarose were previously determined and held constant during fitting. Experimentally recorded MT profile is shown as a scatter plot; theoretical prediction after computational optimization is given as dashed line. The spectral area close to the H_2_O resonance (4.7 ppm) is shown enlarged below. Saturation amplitudes (*B*_1_) are given in Hz. Resulting physical parameters of condensed (5′-CAG-3′)_31_ are given within the plot. Fit deviation indicates overall deviation of experimental datapoints from the model prediction after computational optimization. **c**, Physical parameters of (5′-CAG-3′)_31_ condensates at different concentrations of total RNA, determined via CONDENSE-MT. **d**, Temperature-dependent microscopy of biphasic (5′-CAG-3′)_31_ at varying overall concentrations. Imaging occurred at 55 °C in the presence of 50 mM MgCl_2_. Scale bars, 2 μm. **e**, Schematic representation of the partition coefficient (ratio of RNA fraction in dense and dilute phase) and relative water content per unit of condensed RNA (ratio of *R*_2,water_/*P*_condensate_). **f**, Comparison of normalized population of condensed RNA (obtained via CONDENSE-MT) and normalized *R*_2,water_ (corrected for *R*_2,water_ = 4.13 s^−1^ in absence of (5′-CAG-3′)_31_) on variations in overall concentration of (5′-CAG-3′)_31_. *P*_condensate_ and *R*_2,water_ were normalized to the respective parameter at 37.5 μM total (5′-CAG-3′)_31_. **g**–**i**, CONDENSE-MT analysis of (5′-CAG-3′)_31_ condensates at 50 mM and 30 mM MgCl_2_ as a function of (5′-CAG-3′)_31_ concentration: population of condensed RNA (**g**), *T*_2_ relaxation constant of condensed RNA (**h**) and direct comparison of the population of condensed RNA with the resulting *R*_2,water_ (**i**). **j**, Scheme of (5′-CAG-3′)_31_ partitioning as a function of MgCl_2_ concentration. Partition coefficient of (5′-CAG-3′)_31_ is indicated by the ratio of dilute-phase RNA and the number of schematic RNA condensates. Water molecules and Mg^2+^ ions are schematically indicated by blue and green circles. **b**,**g**–**i**, All errors indicate parameter uncertainties, which were estimated from the diagonal elements of the variance–covariance matrix resulting from fitting experimental data to the CONDENSE-MT model. Error bars are centred around the resulting parameter value after computational optimization ± parameter uncertainty. Source data

**Extended Data Fig. 6:**
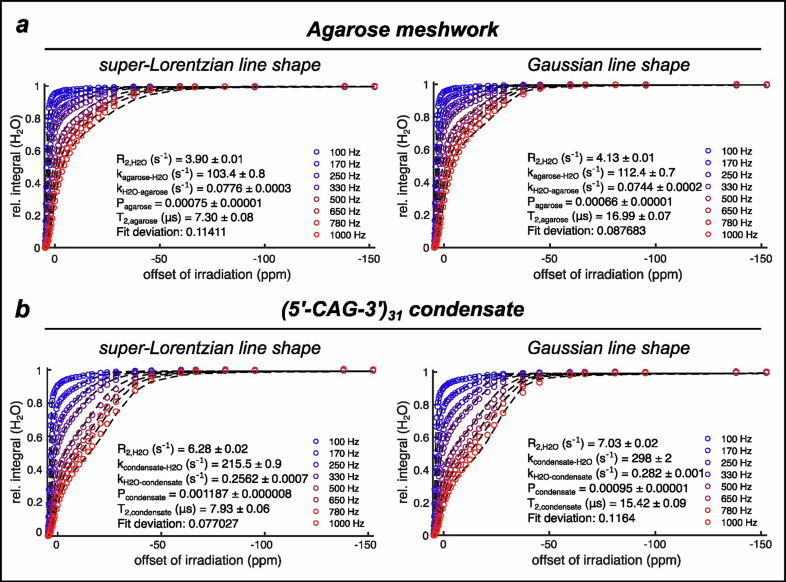
Line shape analysis of agarose and RNA condensate MT effects. **a** Multi-parameter global fitting of the MT effect of 0.5% (w/v) agarose assuming super-Lorentzian line shape (*left*) or Gaussian line shape (*right*) for the semi-solid agarose meshwork. Resulting physical parameters of the agarose meshwork are given in each panel. **b** Multi-parameter global fitting of the MT effect of biphasic (5′-CAG-3′)_31_ (150 μM, at 50 mM MgCl_2_) in presence of 0.5% (w/v) agarose, comparing a super-Lorentzian line shape (*left*) or a Gaussian line shape (*right*) of the semi-solid condensate. Physical parameters of the agarose meshwork were held constant during fitting according to previous quantification of the agarose (shown in **a**). **a** and **b**: Experimental data are shown as scatter plot, fit prediction for each offset saturation amplitude is given as dashed line. Parameter errors were estimated from the diagonal elements of the variance-covariance matrix resulting from fitting experimental data to the CONDENSE-MT model. Fit deviation indicates overall deviation of experimental datapoints from the model prediction after computational optimization. Source data

As anticipated, we could detect the (5′-CAG-3′)_31_ condensates as an additional, concentration-dependent MT pool, centred at approximately 7.8 ppm (Fig. 4a). We determined longitudinal relaxation rates for water in presence of these condensates via inversion recovery to complement input parameters for global fitting (Extended Data Table 1 and Supplementary Note 2), followed by CONDENSE-MT analysis for (5′-CAG-3′)_31_ condensates (Fig. 4b). Unlike agarose, which required a Gaussian line shape for optimal fitting (Supplementary Note 2), we obtained superior fitting results for RNA condensates with a super-Lorentzian line shape (Extended Data Fig. 6a,b). This can be attributed to the partially ordered nature of biochemical condensates and aligns with theoretical considerations for semi-solid biological materials^34,40^. At 150 μM (5′-CAG-3′)_31_, our analysis revealed that a relative proton population of 0.12% contributes to the condensate MT pool, exchanging with the aqueous solvent at an overall exchange rate constant of 215 s^−1^ (Fig. 4b). Given that we expected a proton population of 0.15% considering all protons present in (5′-CAG-3′)_31_ (~1,100 protons, compared with 106 M water protons, therefore $\frac{1,100\times 0.15\,{\mathrm{mM}}}{(2\times 53\times {10}^{3}+1,100\times 0.15)\,{\mathrm{mM}}}\cong 0.15 \%$), this suggests that most RNA is sequestered into the droplets and aligns with the very weak dilute-phase signal under biphasic conditions (Fig. 1a). CONDENSE-MT reported an *R*_2_ rate of 6.3 s^−1^ for bulk water, reflecting the presence of an additional pool of motion-restricted water molecules. CONDENSE-MT revealed a *T*_2_ relaxation constant of 7.8 μs for condensed (5′-CAG-3′)_31_, indicating very slow molecular tumbling of condensed RNA as *T*_2_ relaxation constants for fast-tumbling biomolecules in solution are usually in the order of milliseconds (Fig. 4b)^39,41^. Addition of yet another semi-solid pool to our model did not improve our quantitative analyses, suggesting that the simple model used here describes our (5′-CAG-3′)_31_ system reasonably well (Supplementary Note 3 and Supplementary Fig. 1).

Next, we characterized condensates of (5′-CAG-3′)_31_ at an RNA concentration range between 10 μM and 150 μM (Fig. 4c and Extended Data Table 1). Above 25 μM RNA, we detected populations of condensed RNA that scaled proportionally with total RNA concentration. Similarly, the *R*_2_ relaxation rate of water increased from 4.1 s^−1^ to 6.3 s^−1^, indicating that the amount of condensate-bound water steadily increased with the overall amount of condensed RNA. As expected, the exchange rate constant from water to the condensate, which encodes the population of the MT pool due to chemical equilibrium (Methods) scaled with RNA concentration between 25 μM and 150 μM (5′-CAG-3′)_31_. The reverse exchange rate constant, which, in approximation, corresponds to the overall exchange rate, remained constant. The *T*_2_ relaxation of condensed (5′-CAG-3′)_31_ remained unchanged over a wide range of RNA concentrations, suggesting concentration-independent condensate semi-solid properties. Based on this, we approximated molecular tumbling of condensed RNA to occur at microsecond rotational correlation times and thus 3–4 orders of magnitude slower than expected for free biomolecules in solution, which are typically tumbling at nanosecond rotational correlation times (Supplementary Note 4 and Supplementary Fig. 2). At 10 µM (5′-CAG-3′)_31_, we observed a lower-than-expected MT-pool population, along with a notably elevated *T*_2_ relaxation constant (Fig. 4c). This suggests incomplete condensation due to too low RNA concentration, resulting in altered physical properties of the resulting condensates. We could also observe this effect at 25 µM RNA, albeit to a lesser extent. To visualize the effect of RNA concentration on condensate morphology, we performed temperature-dependent bright-field microscopy of (5′-CAG-3′)_31_ condensates (Fig. 4d). We observed densely packed droplets at high RNA concentrations, whereas fewer droplets were formed per area as the RNA concentration decreased to 37.5 µM. Within this range, the average droplet radius was around 0.5 μm to 1 μm and appeared relatively unaffected by varying RNA concentration, while the number of droplets increased with increased concentrations of overall RNA. These data align with a constant *T*_2_ value of condensate-associated RNA over a wide range of RNA concentrations, indicating constant dynamic properties of condensed RNA. Below 25 µM (5′-CAG-3′)_31_, we observed a marked reduction in droplet size with decreasing RNA concentration, consistent with elevated *T*_2_ relaxation constants and thus increased tumbling rates of condensed RNA under these conditions (Fig. 4c,d). This supports the hypothesis of incomplete condensation, such that the condensates nucleate but are not capable of growing to their final volume due to too little RNA available in solution.

Next, we normalized the population of condensed RNA from these datasets to the corresponding population at 37.5 μM (5′-CAG-3′)_31_, as this is the lowest RNA concentration where we observed full condensation (Fig. 4c). These data revealed a strictly proportional increase in normalized condensate population at increasing overall RNA concentration, which points to a constant partition coefficient $p=\frac{{\mathrm{RNA}}({\mathrm{condensate}})}{{\mathrm{RNA}}({{\mathrm{dilute}\;{\rm{phase}}}})}$ of RNA being sequestered into the condensate (Fig. 4e,f). Furthermore, when normalizing the increase in water *R*_2_ rate with increasing concentration of overall RNA, we again observed strict proportionality (Fig. 4f). Given the sensitivity of CONDENSE-MT to condensed water (Extended Data Fig. 5b), we conclude that the relative water content of a given unit of condensed RNA is not affected by varying RNA concentration. This agrees with constant dynamic properties of condensed RNA and concentration-independent droplet morphology (Fig. 4c,d) across the range of overall RNA concentrations used in our experiments.

### MgCl_2_ modulates partitioning of (5′-CAG-3′)_31_ on condensation

**Extended Data Fig. 7:**
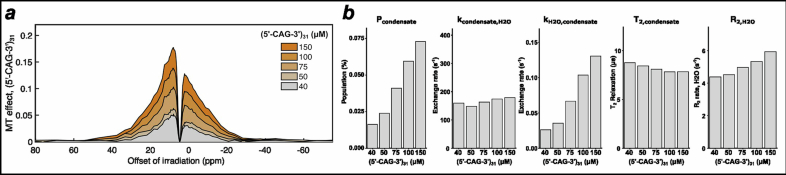
CONDENSE-MT analysis of (5′-CAG-3′)_31_ condensates at 30 mM MgCl_2_. **a** Net MT effect of biphasic (5′-CAG-3′)_31_ at 30 mM MgCl_2_, calculated by subtraction of the corresponding, water-detected MT profiles from respective MT profile of the agarose meshwork (0.5%), at different concentrations of overall RNA at 250 Hz offset saturation. **b** Physical parameters of (5′-CAG-3′)_31_ at 30 mM MgCl_2_ (shown in **a**), determined via CONDENSE-MT. All condensates were embedded in agarose (0.5%, w/v). Physical parameters of the agarose meshwork (Fig. 2b) were held constant during fitting. Source data

**Extended Data Fig. 8:**
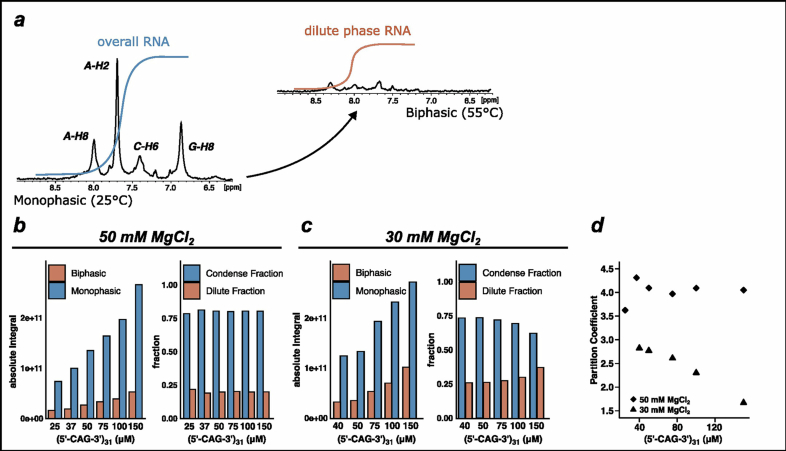
1D ^1^H NMR integral analysis of (5′-CAG-3′)_31_ upon condensation. **a** Spectral area of observable aromatic and amino resonances of (5′-CAG-3′)_31_ (150 μM) under monophasic conditions (25 °C) and biphasic conditions (55 °C) in presence of 0.5% agarose (w/v) and 50 mM MgCl_2_. **b** and **c** Absolute integrals of the spectral area shown in **a** under monophasic (25 °C) and biphasic (55 °C) conditions at different concentrations of overall RNA (*left panel* each*)*. *Right panel:* Fraction of (5′-CAG-3′)_31_ in dense and dilute phase under biphasic conditions, calculated by normalization of the absolute integral under biphasic conditions (dilute phase RNA) to the respective integral under monophasic conditions (total RNA). Condensed Fraction: 1-Dilute Fraction. **d** Partition coefficient of (5′-CAG-3′)_31_ at 50 mM MgCl_2_ (**b**) and at 30 mM MgCl_2_ (**c**) upon phase separation at different RNA concentrations. Partition coefficient was determined by the ratio of fraction of (5′-CAG-3′)_31_ in dense phase and fraction of (5′-CAG-3′)_31_ in dilute phase, shown in **b** and **c**, *right panel* each. Source data

Given the crucial role of MgCl_2_ for RNA condensation in vitro (Extended Data Fig. 1a), we next lowered the MgCl_2_ concentration to 30 mM and characterized the resulting (5′-CAG-3′)_31_ condensates via CONDENSE-MT (Fig. 4g–i and Extended Data Fig. 7a,b). At any given concentration of overall (5′-CAG-3′)_31_, we consistently detected a lower population of condensed RNA at lower MgCl_2_, indicating a lower RNA partition coefficient (Fig. 4g). Increasing the RNA concentration did not result in a linear increase in the amount of condensed RNA but rather to plateau, in contrast to what we observed at 50 mM MgCl_2_, suggesting a decreasing RNA partition coefficient at increasing RNA concentrations. To confirm this independently, we approximated the partition coefficient of (5′-CAG-3′)_31_ on condensation by integrating the aromatic region of ^1^H-NMR spectra of (5′-CAG-3′)_31_ (Extended Data Fig. 8a). This integral is correlated with overall RNA and dilute-phase RNA under monophasic and biphasic conditions, respectively. We estimated the RNA fraction in dilute (${\mathrm{RNA}}_{\mathrm{dilute}}=\frac{\mathrm{Integral}(\mathrm{biphasic})}{\mathrm{Integral}(\mathrm{monophasic})}$) and condensed phase (RNA_condensed_ = 1 − RNA_dilute_) from these integrals and approximated the partition coefficient of (5′-CAG-3′)_31_ on condensation as the ratio of these fractions. Notably, this confirmed both the constant partition coefficient at 50 mM MgCl_2_ and the lower partition coefficient at 30 mM MgCl_2_, the latter of which is further reduced on increasing concentrations of (5′-CAG-3′)_31_ (Extended Data Fig. 8b–d), supporting the results obtained by our water-detected CONDENSE-MT approach (Fig. 4e–g). At 30 mM MgCl_2_, we detected increased RNA dynamics in the condensates already below 50 μM (5′-CAG-3′)_31_ due to the lower population of the RNA condensate, while the dynamic properties of condensed RNA were not MgCl_2_-dependent at higher RNA concentrations (Fig. 4h). Despite differences in RNA partitioning, the correlation between water *R*_2_ rate and condensate population remained unchanged across MgCl_2_ concentrations, suggesting that the relative water content per unit of condensed RNA is not affected by variations in MgCl_2_ concentration (Fig. 4i,j).

### Number of CAG repeats affects condensate hydration

**Fig. 5:**
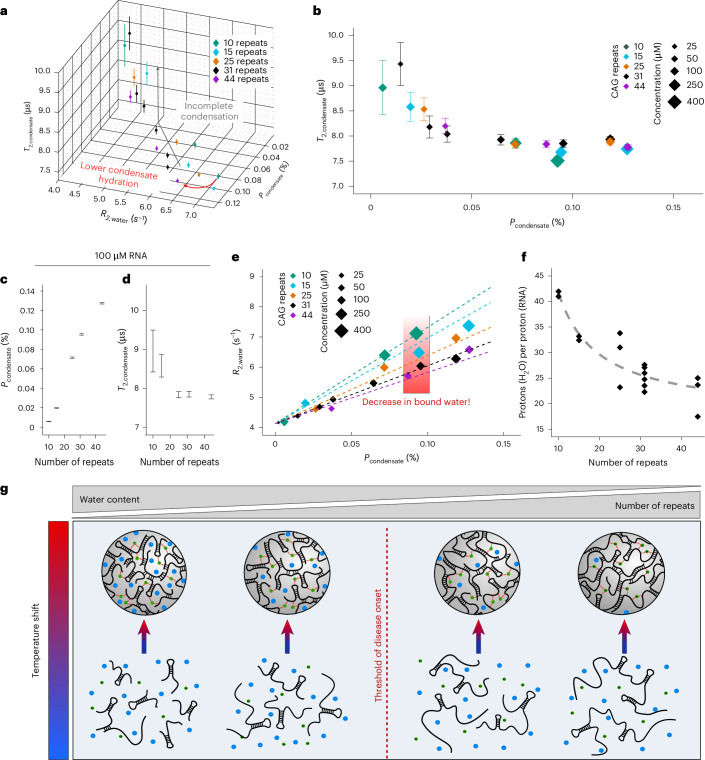
Effect of CAG repeat number on CAG condensates. **a**, Three-dimensional comparison of CAG-condensate parameters (*T*_2,condensate_, *R*_2,water_ and *P*_condensate_) for CAG condensates as a function of CAG repeat number (indicated by colour). Condensates were reconstituted across a variety of molar input concentrations of the respective RNA (Extended Data Table 1). **b**, Comparison of *T*_2,condensate_ with *P*_condensate_ as a function of CAG RNA length. **c**,**d**, Comparison of *P*_condensate_ (**c**) and *T*_2,condensate_ (**d**) as a function of CAG repeat number at 100 μM RNA concentration. **a**–**d**, All parameters were determined using CONDENSE-MT. Error bars indicate parameter uncertainties, estimated from the diagonal elements of the variance–covariance matrix resulting from fitting experimental data to the CONDENSE-MT model, and are centred around the resulting parameter value after computational optimization ± parameter uncertainty. **e**, Direct comparison of $R_{{2,{\rm{H}}_{2}{\rm{O}}}}$ with *P*_condensate_ as a function of RNA length. Linear trendlines indicate increase of $R_{{2,{\rm{H}}_{2}{\rm{O}}}}$ as function of *P*_condensate_. Datapoints with *P*_condensate_ < 0.025% were excluded for the trendline. **f**, Ratio of protons (H_2_O/RNA) in CAG condensates as a function of RNA length, obtained from individual calculation of the proton ratio (H_2_O/RNA) across multiple *P*_condensate_ datasets (shown in **a**) for each RNA length, varying in their molar RNA concentration. Calculations were performed using all datasets with a *P*_condensate_ > 0.025%. Lower water content at increasing RNA length is indicated by dashed trendline. Detailed information is given in the Supplementary Note 5. **g**, Scheme of RNA condensation as a function of the number of CAG repeats. Variations in water content of RNA condensates is indicated by the number of water symbols (blue circles) per RNA droplet. Mg^2+^ is schematically indicated by green dots. Source data

**Extended Data Fig. 9:**
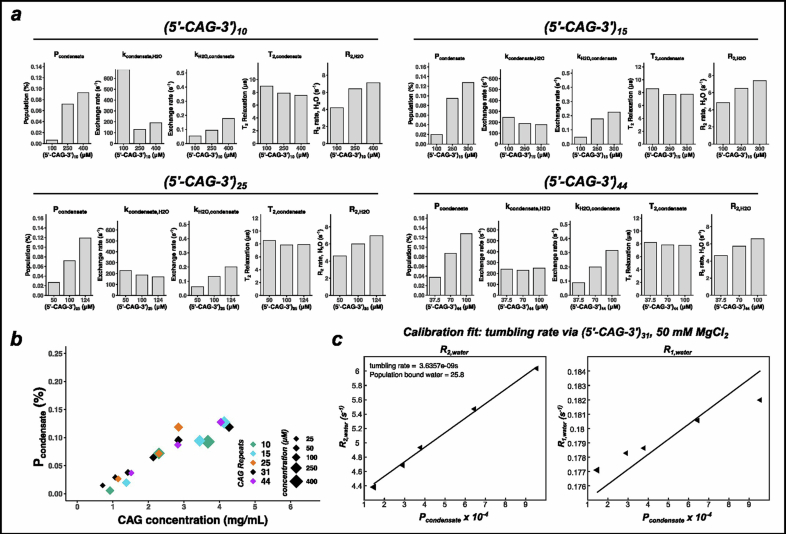
Condensation of CAG repeat-expansion RNA at varying CAG repeat number. **a** Characterization of CAG repeat-expansion RNA condensates via CONDENSE-MT as a function of number of CAG repeats and molar concentration for CAG repeat RNA. All data, including the associated errors, are also listed in Extended Data Table 1. **b** Population of condensed RNA, detected via CONDENSE-MT (shown in **a**) as a function of RNA concentration (mg/mL) for CAG repeat RNA at varying number of CAG repeats. Molar concentration is indicated by marker size, CAG repeat number is indicated by color. **c** Global fit for the tumbling rate of condensed water via R_2,water_ (obtained from CONDENSE-MT, Fig. 4) and R_1,water_ (measured via inversion recovery) as a function of P_condensate_ of condensed (5′-CAG-3′)_31_ (at molar concentrations ranging from 37.5 μM to 150 μM, determined in Fig. 4), based on dipole-dipole nuclear spin relaxation (Bloembergen-Purcell-Pound theory)^39,41,43^. The obtained tumbling rate of water bound and the corresponding population of water-protons in a (5′-CAG-3′)_31_ condensate are given in the upper left. A detailed description of the formulas used for this fit is given in the Supplementary Note 5. Source data

The number of CAG repeats is crucial for disease onset and in-cell foci formation^3,6^. Therefore, we next investigated the effect of CAG repeat number on condensate properties, by characterizing condensates of CAG RNA with 10 to 44 CAG repeats via CONDENSE-MT (Fig. 5a, Extended Data Fig. 9a and Extended Data Table 1). Here, the overall population of condensed RNA was strictly proportional to the mass concentration of RNA present in solution, suggesting that the partition coefficient of RNA on condensation is not affected by RNA length (Extended Data Fig. 9b). As before, we detected increased dynamic properties of condensed RNA once the population of condensed RNA was below a threshold of approximately 0.025%, which we attribute to incomplete condensation due to the limited amount of RNA available in solution (Fig. 5a,b). Once sufficient RNA was available for full dynamic arrest, the tumbling rate of condensed RNA approached a steady value, independent of the number of repeats per oligomer (Fig. 5b). Consequently, when fixing the molar concentration to 100 μM, we observed full dynamic arrest for CAG oligomers with a repeat number of more than 25, while shorter RNA was not capable of full condensation, suggesting that longer CAG RNA is more potent to achieve condensate formation due to a higher concentration of RNA repeats (Fig. 5c,d). Under conditions where we observed full condensation, we detected a pronounced decrease in water *R*_2_ rate at increasing number of repeats (Fig. 5a,e). While, for instance, (5′-CAG-3′)_10_ at a population of 0.093% elevated the *R*_2_ rate of bulk water to 7.12 s^−1^, leading to a net effect of 2.99 s^−1^ additional *R*_2,water_ compared with the agarose meshwork (Fig. 3b), the effect on *R*_2,water_ decreased steadily with increasing RNA length at similar condensate populations. Accordingly, condensed (5′-CAG-3′)_44_ at a similar population of 0.087% elevated *R*_2,water_ by only an additional 1.58 s^−1^ (Fig. 5e, red box). This suggests a lower water content in the condensates formed by longer CAG repeat-expansion RNA^37,39,41^.

Finally, the proportionality between water *R*_2_ rates and the amount of condensed (5′-CAG-3′)_31_ (Fig. 4f,i) is reminiscent of previous reports, showing that water bound to large biomolecules linearly elevates the bulk water relaxation rates depending on the fraction of bound water and its rotational correlation time^39,42,43^. Therefore, we used the water relaxation rates in presence of (5′-CAG-3′)_31_ condensates and the established Bloembergen–Purcell–Pound relaxation formalism^39,41–43^, and estimated a rotational correlation time of 3.6 ns for condensate-bound water and an excess of 26 water protons per proton of condensed RNA (Extended Data Fig. 9c). This suggests that water in the condensate tumbles at rotational correlation times approximately three orders of magnitude slower than free water in solution. For detailed calculations, we refer to Supplementary Note 5 and Supplementary Fig. 3a,b. Carrying out these analyses on our datasets of CAG RNA at different length (Fig. 5a,b,e) further revealed that the relative water content within CAG condensates decreases from roughly 42 protons (21 water molecules) to 22 protons (11 water molecules) per proton of RNA when increasing the RNA length from 10 to 44 CAG repeats (Fig. 5f and Supplementary Fig. 3c). Thus, a fourfold increase in RNA length induces a reduction of water molecules per RNA proton in the condensate by approximately 50%, suggesting that longer repeat-expansion RNA forms dryer and RNA-denser condensates (Fig. 5g).

## Discussion

Here, we introduced a label-free and non-invasive methodological approach, CONDENSE-MT, enabling detection and quantification of biological condensates that are entirely invisible to conventional NMR spectroscopy. CONDENSE-MT is based on solvent detection and therefore applicable at all field strengths, does not demand cryoprobes or isotope labelling and yields signal amplification of two to three orders of magnitude. We therefore anticipate CONDENSE-MT to be of highest interest to research groups interested in biomolecular phase transitions without state-of-the-art NMR equipment or expertise. The physical properties of NMR-invisible condensates are transferred to bulk water only by proton exchange, such that CONDENSE-MT is also applicable in the absence of physical exchange of biomolecules between the dense and dilute phases. By this, we have overcome limitations of established NMR methods based on detection of the biomolecule of interest^31^. It may be noted that our solvent-detected approach results in integrated information about the entire condensed biomolecule and is therefore not capable of single-residue resolution. Given the repetitive nature and the resulting, regular folding pattern of CAG RNA (Extended Data Fig. 2), this is a negligible limitation for repeat-expansion RNA condensed systems. Furthermore, the presence of agarose MT effects can limit the capability of the method to detect RNA condensates at very low RNA levels. Using CONDENSE-MT, we confirmed slow dynamics within the condensate as previously postulated due to the capability of repeat-expansion RNA for multivalent base pairing and network formation^3,12^, leading to rotational correlation times in the order of microseconds (Fig. 4b,c and Supplementary Note 4). We showed that RNA partitioning is strongly dependent on MgCl_2_ levels (Fig. 4e–g,j), suggesting that heterotypic interactions between RNA and Mg^2+^ are the key driving force for condensation of (5′-CAG-3′)_31_ (refs. ^44–46^). Finally, CONDENSE-MT enabled us to detect the hydration state of condensed RNA, which might contribute to understanding RNA foci formation in the context of RNA repeat-expansion diseases. We speculate that the increase in RNA density due to the strongly reduced water content in RNA condensates might enable stable foci formation in a high-density nuclear environment in vivo for RNA above a specific threshold number of repeats. In addition, an increased RNA-to-water mass fraction for higher CAG repeat number could promote neurotoxic liquid-to-solid maturation of the condensate over time^7,13^. Although we used high-temperature conditions to induce condensation of (5′-CAG-3′)_31_ in vitro, we expect the LCST to be substantially lower in highly crowded and viscous eukaryotic nuclei. However, whether neurotoxicity of RNA foci may occur due to liquid-to-solid maturation of the condensate, sequestration of RNA-binding proteins to the foci or another mechanism requires further investigation^5,7,9,47^.

In future studies, the broad applicability of CONDENSE-MT can provide pivotal insights into the physicochemical properties of biomolecular condensates in general, including disease-associated protein condensates as well as further detailed characterization of RNA condensates as a function of a variety of biochemical conditions or in the presence of therapeutic agents targeting neurotoxic RNA aggregates. Efforts are underway to adapt the methodology to species-specific characterization of multicomponent condensates. Given the sensitive, water-detected readout of our experiment, we consider CONDENSE-MT to be a beneficial tool also in future drug-screening campaigns involving RNA condensates with the potential to be translated even to in-cell studies.

## Methods

### Theory

Since the concentration of both MT pools present in our solution is very small relative to the liquid pool, we neglected direct proton exchange between the two MT pools. Equilibrium exchange between an MT pool and solvent H_2_O is described via the product of a concentration-independent exchange rate constant and the relative population of protons contributing to the MT pool or solvent H_2_O:

1 $${P}_{\mathrm{MT}}\times {k}_{{\mathrm{MT}},{\mathrm{H}}_2{\rm{O}}}={P}_{{\mathrm{H}}_2{\rm{O}}}\times {k}_{{{\mathrm{H}}_2{\rm{O}}},{\mathrm{MT}}}$$

The overall rate of exchange, defined as the overall exchange rate constant $k_{{\rm{ex}}}\,=\,k_{{\rm{MT}},{{\rm{H}}_2{\rm{O}}}}\,+\,k_{{{\rm{H}}_2{\rm{O}},{\rm{MT}}}}$, can be approximated by $k_{{\rm{MT},{H_2{\rm{O}}}}}$ since ${P}_{\mathrm{MT}}\,\ll{P}_{{\mathrm{H}}_2{\rm{O}}}$ (refs. ^23,24,34^). The population of an MT pool is given by

2 $${P}_{\mathrm{MT}}=\frac{{k}_{{\mathrm{H}}_2{\rm{O}},{\mathrm{MT}}}}{{k}_{{\mathrm{H}}_2{\rm{O}},{\mathrm{MT}}}+{k}_{{\mathrm{MT}},{\mathrm{H}}_2{\rm{O}}}}$$

According to refs. ^35,36^, the relative magnetization of the detected solvent H_2_O (*Z*), as a function of irradiation at an offset of Δ*ω* (rad s^−1^) with amplitude *ω*_1_ = *γB*_1_ (rad s^−1^), can generally be described using the corresponding rotating-frame longitudinal relaxation of the detected nuclei (*R*_1*ρ*_(Δ*ω*)):

3 $$Z\left(\Delta {{\omega}}\right)=\frac{{\Delta {{\omega}}}^{2}\times {R}_{1,{\mathrm{H}}_2{\rm{O}}}}{\left({\Delta {{\omega}}}^{2}+{{{\omega}}}_{1}^{2}\right)\times {R}_{1 {\rho}}(\Delta {{\omega}})}$$

The overall *R*_1*ρ*_ of our system, affected by offset irradiation Δ*ω*, can then be derived as a linear combination of the underlying rotating-frame relaxation of H_2_O in isolation (${R}_{1{{\rho}}({\mathrm{H}}_2{\rm{O}})}$) and additional, exchange relayed relaxation originating from the semi-solid MT pools (*R*_MT_). Since semi-solid MT pools were shown to affect longitudinal recovery of bulk water in a concentration-dependent manner^36^, we determined the apparent longitudinal relaxation rate of water (${R}_{1,{\mathrm{H}}_2{\rm{O}}}$) via inversion recovery for each sample. According to these considerations, we modelled our system in presence of an agarose meshwork by:

4a $${R}_{1{\rho}}\left(\Delta {{\omega}}\right)={R}_{1{\rho}({\mathrm{H}}_2{\rm{O}})}+{R}_{\mathrm{agarose}}$$

For quantifying the RNA condensate embedded in an agarose meshwork, we assumed:

4b $${R}_{1{{\rho}}}\left(\Delta {{\omega}}\right)={R}_{1{{\rho}}({\mathrm{H}}_2{\rm{O}})}+{R}_{\mathrm{agarose}}+{R}_{\mathrm{condensate}}$$

Here, our superimposed *R*_1*ρ*_ (Δ*ω*) description slightly differs from the validated model from ref. ^36^, as we omit the presence of a liquid-state CEST pool while assuming the presence of two independent, physically different semi-solid MT pools, to enable selective characterization of two MT pools (agarose and RNA condensate) in a stepwise process using equations (4a) and (4b). The rotating-frame longitudinal relaxation of bulk H_2_O in isolation (${R}_{1{\rho}({\mathrm{H}}_2{\rm{O}})}$) depends only on saturation offset and amplitude:

5 $${R}_{1{{\rho}}\left({\mathrm{H}}_2{\rm{O}}\right)}={R}_{1,{\mathrm{H}}_2{\rm{O}}}\frac{{\Delta {\rm{\omega }}}^{2}}{\left({\Delta {\rm{\omega }}}^{2}+{{\rm{\omega }}}_{1}^{2}\right)}+{R}_{2,{\mathrm{H}}_2{\rm{O}}}\frac{{{\rm{\omega }}}_{1}^{2}}{\left({\Delta {\rm{\omega }}}^{2}+{{\rm{\omega }}}_{1}^{2}\right)}$$

The exchange relayed contributions to H_2_O relaxation by a semi-solid MT pool (*R*_agarose_ and *R*_condensate_) are generally expressed via^36^:

6 $$\begin{array}{c}{R}_{\mathrm{MT}}=\displaystyle\frac{\left(\Delta {{\rm{\omega }}}_{{{\mathrm{H}}_2{\mathrm{O}}}}^{2}+{\left({R}_{2,{{\mathrm{H}}_2{\mathrm{O}}}}-{R}_{1{{\rho }}\left({{\mathrm{H}}_2{\mathrm{O}}}\right)}\right)}^{2}\right)\left({k}_{{\mathrm{MT}},{{\mathrm{H}}_2{\mathrm{O}}}}\times \left({R}_{1,{{\mathrm{H}}_2{\mathrm{O}}}}-{R}_{1{{\rho }}\left({{\mathrm{H}}_2{\mathrm{O}}}\right)}\right)+\left({R}_{1,{\mathrm{MT}}}{+R}_{\mathrm{rfMT}}-{R}_{1{{\rho }}\left({{\mathrm{H}}_2{\mathrm{O}}}\right)}\right)\left({k}_{{{\mathrm{H}}_2{\mathrm{O}}},{\mathrm{MT}}}+\left({R}_{1,{{\mathrm{H}}_2{\mathrm{O}}}}-{R}_{1{{\rho }}\left({{\mathrm{H}}_2{\mathrm{O}}}\right)}\right)\right)\right)}{(\Delta {{\rm{\omega }}}_{{\mathrm{H}}_2{\mathrm{O}}}^{2}+{\left({R}_{2,{{\mathrm{H}}_2{\mathrm{O}}}}-{R}_{1{{\rho }}\left({{\mathrm{H}}_2{\mathrm{O}}}\right)}\right)}^{2})({k}_{{\mathrm{MT}},{{\mathrm{H}}_2{\mathrm{O}}}}+{k}_{{{\mathrm{H}}_2{\mathrm{O}}},{\mathrm{MT}}}+{R}_{1,{{\mathrm{H}}_2{\mathrm{O}}}}+{R}_{1,{\mathrm{MT}}}+{R}_{\mathrm{rfMT}}-{2R}_{1{{\rho }}\left({{\mathrm{H}}_2{\mathrm{O}}}\right)})}\\ +\displaystyle\frac{{{\rm{\omega }}}_{1}^{2}({R}_{2,{{\mathrm{H}}_2{\mathrm{O}}}}-{R}_{1{{\rho }}\left({{\mathrm{H}}_2{\mathrm{O}}}\right)})\left({k}_{{\mathrm{MT}},{{\mathrm{H}}_2{\mathrm{O}}}}+\left({R}_{1,{\mathrm{MT}}}+{R}_{\mathrm{rfMT}}-{R}_{1{{\rho }}\left({{\mathrm{H}}_2{\mathrm{O}}}\right)}\right)\right)}{(\Delta {{\rm{\omega }}}_{{\mathrm{H}}_2{\mathrm{O}}}^{2}+{\left({R}_{2,{{\mathrm{H}}_2{\mathrm{O}}}}-{R}_{1{{\rho }}\left({{\mathrm{H}}_2{\mathrm{O}}}\right)}\right)}^{2})({k}_{{\mathrm{MT}},{{\mathrm{H}}_2{\mathrm{O}}}}+{k}_{{{\mathrm{H}}_2{\mathrm{O}}},{\mathrm{MT}}}+{R}_{1,{{\mathrm{H}}_2{\mathrm{O}}}}+{R}_{1,{\mathrm{MT}}}+{R}_{\mathrm{rfMT}}-{2R}_{1{{\rho }}\left({{\mathrm{H}}_2{\mathrm{O}}}\right)})}\\ \begin{array}{c}+2\left({R}_{2,{{\mathrm{H}}_2{\mathrm{O}}}}-{R}_{1{{\rho }}\left({{\mathrm{H}}_2{\mathrm{O}}}\right)}\right)\left({k}_{{\mathrm{MT}},{{\mathrm{H}}_2{\mathrm{O}}}}\left({R}_{1,{{\mathrm{H}}_2{\mathrm{O}}}}-{R}_{1{{\rho }}\left({{\mathrm{H}}_2{\mathrm{O}}}\right)}\right)+\left({R}_{1,{\mathrm{MT}}}{+R}_{\mathrm{rfMT}}-{R}_{1{{\rho }}\left({{\mathrm{H}}_2{\mathrm{O}}}\right)}\right)\left({k}_{{{\mathrm{H}}_2{\mathrm{O}}},{\mathrm{MT}}}+\left({R}_{1,{{\mathrm{H}}_2{\mathrm{O}}}}-{R}_{1{{\rho }}\left({{\mathrm{H}}_2{\mathrm{O}}}\right)}\right)\right)\right)\\ +{{\rm{\omega }}}_{1}^{2}({R}_{2,{{\mathrm{H}}_2{\mathrm{O}}}}+{k}_{{\mathrm{MT}},{\mathrm{H-2O}}}+{R}_{1,{\mathrm{MT}}}+{R}_{\mathrm{rfMT}}-{2R}_{1{{\rho }}\left({{\mathrm{H}}_2{\mathrm{O}}}\right)})\end{array}\end{array}$$

Given the exclusive presence of only MT pools in our model system, this expression aligns with formulations derived from Bloch–McConnell equations by Henkelmann et al. under steady-state conditions, as previously demonstrated^24,36^. The loss of longitudinal magnetization of a semi-solid MT pool due to radio frequency off-resonance saturation, which is relayed to the observed bulk H_2_O via chemical exchange, is represented by *R*_rfMT_. This term encodes the physical properties of the broad, invisible resonance of the MT pool by introducing line width (via *T*_2_ relaxation of the MT pool), line shape and the chemical shift centre of the MT pool (Δ*ω*_MT_) into the *Z*(Δ*ω*) framework. The effect of off-resonance saturation on semi-solids at Gaussian line shape is commonly described as:

7a $${R}_{\mathrm{rfMT}}={{{\omega}}}_{1}^{2}\sqrt{\displaystyle\frac{{{\uppi}}}{2}}{T}_{2,{\mathrm{MT}}}{\mathrm{e}}^{\displaystyle\frac{{(\Delta {{{\omega }}}_{\mathrm{MT}}{T}_{2,{\mathrm{MT}}})}^{2}}{2}}$$

For materials with super-Lorentzian line shape, *R*_rfMT_ can be defined as:

7b $${R}_{\mathrm{rfMT}}={{{\omega }}}_{1}^{2}{{\uppi }}{\int }_{0}^{\displaystyle\frac{{{\uppi }}}{2}}\left(\sin {{\theta }}\sqrt{\displaystyle\frac{{{\uppi }}}{2}}\displaystyle\frac{{T}_{2,{\mathrm{MT}}}}{\left|3{\cos }^{2}\left({{\theta }}\right)-1\right|}{\mathrm{e}}^{-2\left({\displaystyle\frac{\Delta {{{\omega }}}_{\mathrm{MT}}{T}_{2,{\mathrm{MT}}}}{\left|3{\cos }^{2}\left({{\theta }}\right)-1\right|}}\right)^{2}}\right){\mathrm{d}}{{\theta }}$$

The angle *θ* reflects the extent of dipolar interactions in the MT pool and thus accounts for partially ordered, semi-solid material properties of biological condensates, resulting in super-Lorentzian line shape. Thus, *R*_rfMT_ for super-Lorentzian line shapes differs from the Gaussian case when *θ* ≠ 0 (refs. ^24,34,36^).

Given the above definitions, the final MT profile under steady-state conditions (*Z*(Δ*ω*), equation (3)) can be optimized computationally via global fitting using the definitions shown in equations (4)–(7). Thus, experimental MT profiles were globally fitted to equation (3) via computational optimization of $k_{{\rm{MT}},{{\rm{H}}_2{\rm{O}}}}$, $k_{{{\rm{H}}_2{\rm{O}},{\rm{MT}}}}$, *T*_2,MT_ and ${R}_{2,{\mathrm{H}}_2{\rm{O}}}$ with the separately determined ${R}_{1,{\mathrm{H}}_2{\rm{O}}}$ being held constant during computational optimization (section ‘Data processing and multi-parameter global fitting of MT profiles’). As previously reported, this model is only weakly dependent on *R*_1,MT_, which therefore cannot be precisely estimated by this approach. Per convention, this parameter is therefore set to 1 s^−1^ (refs. ^23,34,48^).

### RNA synthesis

Here (5′-CAG-3′)_31_ RNA was transcribed in vitro from linear DNA templates using recombinant T7 RNA polymerase available in-house. Before in vitro transcription, the T7 promotor region of the DNA templates as annealed with a short complementary DNA fragment to generate the double-stranded promotor region. In vitro transcription occurred for 6–8 h at 37 °C in presence of 6 mM rNTP and 30 mM MgCl_2_. The product mixture was cleared with EDTA, filtered and then purified via ion-exchange chromatography (DNAPac-PA100, 22 × 250, ThermoScientific) under denaturing conditions (6 M urea, 12.5 mM Tris and HCl, pH 7.4, 85 °C). Excess mononucleotides were removed from (5′-CAG-3′)_31_ product by 15% elution buffer (6 M urea, 12.5 mM Tris and HCl, pH 7.4, 0.5 M NaClO_4_). Elution of product RNA occurred via a gradient up to 75% elution buffer. Peak fractions containing (5′-CAG-3′)_31_ were identified via denaturing urea polyacrylamide gel electrophoresis (PAGE), pooled and product RNA was extracted via butanol extraction. Extracted RNA pellets were washed three times by dissolving in RNase-free H_2_O followed by re-extraction with butanol. Final RNA was dissolved in RNase-free H_2_O, lyophilized and stored at −20 °C.

### Optical density assay

Optical density assays were performed at 40 μM (5′-CAG-3′)_31_ in a buffer containing the respective MgCl_2_ concentration and 25 mM Tris and HCl, pH 7.4 (ND-1000 NanoDrop Spectrophotometer, ThermoFisher Scientific). The mixture was tempered in a heat block device before measuring light scattering at 600 nm. All data were acquired in three independently prepared replicates.

### Microscopy

Temperature-dependent imaging was performed using VAHEAT temperature control units (Interherence) mounted into an ECLIPSE Ti2 microscope (Nikon Instruments Inc.) equipped with an ORCA-FusionBT digital camera (Hamamatsu Photonics). Next, 50 μl of sample (at 50 mM MgCl_2_ and 25 mM Tris and HCl, pH 7.4) were loaded and heated to 55 °C at 0.5 °C s^−1^. Bright-field images (×100 magnification) were taken after incubation on 55 °C for 1 min. Images were processed using ImageJ software (Fiji processing package).

### Condensate reconstitution in agarose

Here (5′-CAG-3′)_31_ samples for NMR measurements were prepared in presence of 0.5% low-melting agarose (ThermoFisher Scientific) in 5% (v/v) D_2_O and 25 mM Tris and HCl, pH 7.4. For this, the previously dissolved agarose stock solution (2%, w/v) was molten (95 °C, 10–15 min), cooled to 37 °C and then added as the last reagent into the NMR sample to an end concentration of 0.5% (w/v). The sample was transferred into the NMR tube after addition of agarose, followed by incubation at room temperature for 15–20 min to solidify the agarose before NMR analyses at room temperature. NMR experiments were always first performed directly at 25 °C before temperature shift to 55 °C, extending the incubation time at room temperature to 3 h overall.

### NMR data acquisition

NMR data were acquired in 3-mm NMR sample tubes on a 700-MHz Avance NEO spectrometer equipped with He-cooled cryoprobes. Data were recorded and processed with TopSpin v.4 software (Bruker). One-dimensional (1D) NMR spectra for RNA detection were recorded using water suppression (excitation sculpting, zgesgp pulse sequence)^49^, centred at 4.7 ppm at a spectral width of 22 ppm. For water-detected CEST and MT experiments, spectra were centred at 4.7 ppm. Selective offset saturation was performed using continuous-wave irradiation at *B*_1_ amplitudes of [100, 170, 250, 330, 500, 650, 780, 1,000] Hz using 92 (both sides) or 46 (only upfield saturation of H_2_O) distributed frequency offsets in a range between the ±100 kHz spectral distance to the solvent H_2_O resonance frequency. We applied off-resonance saturation for 5 s, followed by detection of solvent H_2_O spectra by small tip angle excitation (*p* = 1 μs). We used a recovery delay of 4 s to ensure steady-state conditions. CONDENSE-MT data were recorded using two scans per offset saturation frequency and used a phase cycle on the excitation pulse [0 2] to minimize artefacts due to radiation dumping effects.

Longitudinal relaxation of solvent H_2_O was determined for each sample via in-house inversion recovery experiments that used a weak 0.045 G cm^−1^ gradient along the *z* axis during the recovery period to suppress radiation damping effects. We recorded 24 distinct recovery delays up to 15 s and determined the *T*_1_ relaxation constant with self-written MATLAB scripts.

### Data processing and multi-parameter global fitting of MT profiles

Recorded CEST and MT data were first processed with TopSpin, v.4.4.0 (zerofilling, qsine window function, Fourier transformation, phase correction). Subsequent data processing and fitting was performed using MATLAB (version 2022b, MathWorks). For global fitting of experimental data to our model, we optimized the parameters $k_{{\rm{MT}},{{\rm{H}}_2{\rm{O}}}}$, $k_{{{\rm{H}}_2{\rm{O}},{\rm{MT}}}}$, *T*_2,MT_ and ${R}_{2,{\mathrm{H}}_2{\rm{O}}}$ according to the equations described previously^24,36^ while setting ${R}_{1,{\mathrm{H}}_2{\rm{O}}}$ to a constant value determined via inversion recovery (Extended Data Table 1). Since minor variations of the chemical shift centre did not affect the resulting parameters after computational optimization due to the very large line width of semi-solid species, we quantified RNA condensates while centring RNA condensates to the reference frequency of water for simplicity. Global fitting was performed in MATLAB using the implemented, nonlinear fitting tool fminsearch, which applied least-squares regression to the difference between model prediction and experimentally recorded datapoints. To estimate parameter uncertainties, the first-order partial derivatives for the Jacobian matrix were numerically calculated using MATLAB scripts available in-house. From this, the variance–covariance matrix was derived and parameter uncertainties were estimated from the square root of diagonal elements of the variance–covariance matrix. Thus, the errors we report from our computational optimization reflect on how well individual parameters are constrained by experimental data. Furthermore, since the variance–covariance matrix was derived from the Jacobian matrix, small errors indicate that parameter optimization occurred unambiguously without interfering with optimization of the other parameters that were varied during global, iterative optimization.

### Oligonucleotide sequences

**Table:** 

**(5**′**-CAG-3**′**)**_**10**_	5′-CAGCAGCAGCAGCAGCAGCAGCAGCAGCAG-3′
**(5**′**-CAG-3**′**)**_**15**_	5′-CAGCAGCAGCAGCAGCAGCAGCAGCAGCAGCAGCAGCAGCAGCAG-3′
**(5**′**-CAG-3**′**)**_**25**_	5′-CAGCAGCAGCAGCAGCAGCAGCAGCAGCAGCAGCAGCAGCAGCAGCAGCAGCAGCAGCAGCAGCAGCAGCAGCAG-3′
**(5**′**-CAG-3**′**)**_**31**_	5′-CAGCAGCAGCAGCAGCAGCAGCAGCAGCAGCAGCAGCAGCAGCAGCAGCAGCAGCAGCAGCAGCAGCAGCAGCAGCAGCAGCAGCAGCAGCAG-3′
**(5**′**-CAG-3**′**)**_**44**_	5′-CAGCAGCAGCAGCAGCAGCAGCAGCAGCAGCAGCAGCAGCAGCAGCAGCAGCAGCAGCAGCAGCAGCAGCAGCAGCAGCAGCAGCAGCAGCAGCAGCAGCAGCAGCAGCAGCAGCAGCAGCAGCAGCAGCAG-3′

### Reporting summary

Further information on research design is available in the Nature Portfolio Reporting Summary linked to this article.

## Online content

Any methods, additional references, Nature Portfolio reporting summaries, source data, extended data, supplementary information, acknowledgements, peer review information; details of author contributions and competing interests; and statements of data and code availability are available at 10.1038/s41557-025-01968-9.

## Supplementary information

Supplementary Information Supplementary Notes 1–5 and Figs. 1–3.

Reporting Summary

## Source data

Source Data Figs. 1–5 and Extended Data Figs. 1, 4 and 6–9 and Table 1 Numerical source data for Figs. 1–5 and Extended Data Figs. 1, 4 and 6–9 and Table 1; uncropped images for Fig. 4 and Extended Data Fig. 1.

## Figures and Tables

**Extended Data Table 1:**
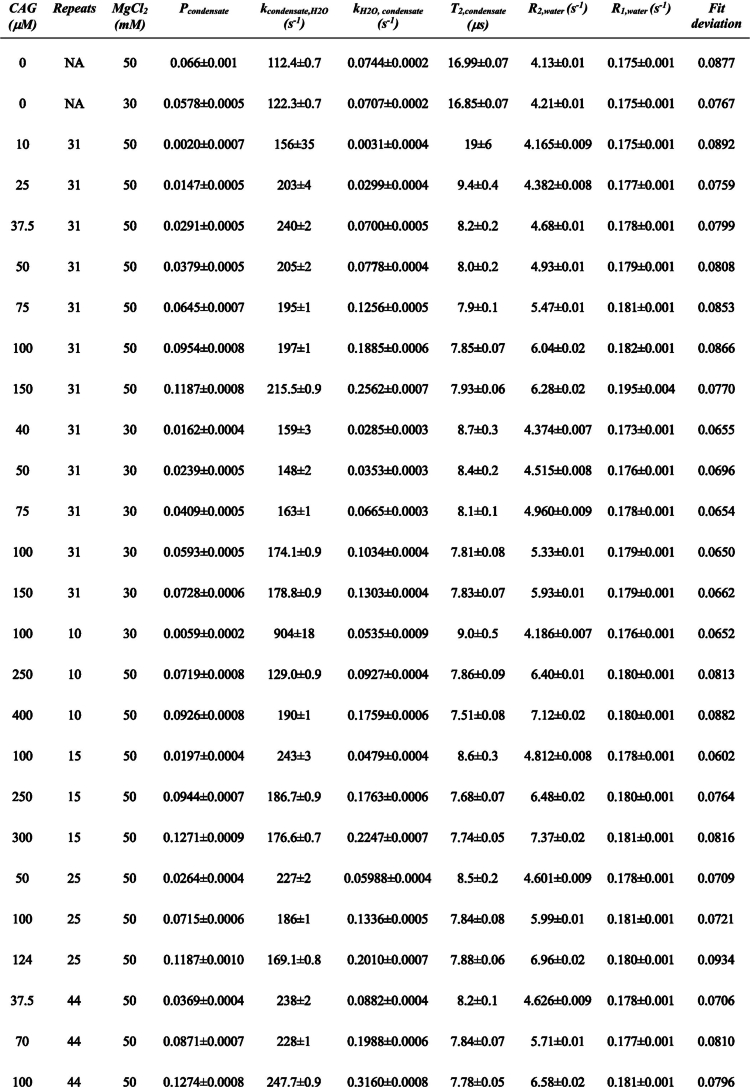
Physical parameters of repeat-expansion CAG condensates

Combined CONDENSE-MT results of CAG condensates across RNA concentrations, number of CAG repeats and MgCl_2_ concentration. All experiments were recorded in presence of agarose 0.5% (w/v) and at 55 °C. Physical parameters of the agarose meshwork, in absence of RNA, at two different MgCl_2_ concentrations are given in the first two rows. Fit deviation represents the overall deviation of all experimental datapoints after computational optimization. Individual parameter uncertainties were estimated from the diagonal elements of the variance-covariance matrix resulting from fitting experimental data to the CONDENSE-MT model. Longitudinal relaxation rates of H_2_O were determined via inversion recovery and held constant during CONDENSE-MT analyses.

Source data

## Data Availability

Source data for all figures (except 1D and two-dimensional (2D) NMR spectra) are provided with this paper. All NMR data, including 1D and 2D NMR spectra, are available via Zenodo at 10.5281/zenodo.15789697 (ref. ^50^).

## Code availability

MATLAB codes used for processing and fitting of NMR data are in the Zenodo repository at 10.5281/zenodo.15789697 (ref. ^50^).
