# Supplementary material for: Water-detected NMR allows dynamic observations of repeat-expansion RNA condensates
Source: Nat Chem. 2025 Oct 15;17(11):1785–94. doi: 10.1038/s41557-025-01968-9 (PMC12580330; doi:10.1038/s41557-025-01968-9)
Supplement: Supplementary file 1 — Supplementary Notes 1–5 and Figs. 1–3. [file 41557_2025_1968_MOESM1_ESM.pdf]

nature chemistry

Supplementary information https://doi.org/10.1038/s41557-025-01968-9

Water-detected NMR allows dynamic observations of repeat-expansion RNA condensates

In the format provided by the authors and unedited

Table of Contents

Supplementary Note 1: Exploiting signal amplifications by water detection............... 2

Supplementary Note 2: Interpretation of CONDENSE-MT results on agarose 2

Supplementary Note 3: Testing for the required model complexity............................ 3

Supplementary Note 4: Approximating tumbling rates of condensed RNA 4

Supplementary Note 5: Approximating condensed water populations in RNA condensates.. 7

References 10

Supplementary Note 1: Exploiting signal amplifications by water detection

Due to the very high concentration of H_2O in biochemical solutions (55 M), our water-detected approach offered substantial sensitivity enhancements due to the continuous transfer of saturated protons towards bulk water during the prolonged offset irradiation (5 s, see main text, Methods). At a concentration of 150 µM (5'-CAG-3')$_{31}$, we detected water attenuation up to 25% of the total water resonance intensity (main text, Fig. 2d). This corresponds to 26 M protons, assuming 53 M bulk water (at 5% D_2O) in solution and two protons per water molecule. Therefore, given 150 µM (5'-CAG-3')$_{31}$ and approximately 1,100 protons per (5'-CAG-3')$_{31}$ oligomer, our approach yields up to a 150-fold signal amplification compared to direct detection of(5'-CAG-3')$_{31}$, which is in addition NMR-invisible in the condensed state.

Supplementary Note 2: Interpretation of CONDENSE-MT results on agarose

We tested our quantitative setup by characterizing the agarose meshwork, as these parameters are required to subsequently quantify semi-solid RNA condensates. Quantitative MT studies, based on water detection, generally yield the ratio of longitudinal and transverse relaxation of bulk water as a unique parameter.[1,2] Therefore, unambiguous quantification of solvent transverse relaxation requires initial knowledge about the longitudinal relaxation rate ($R_{1,H2O}$). To complement the input parameters for global fitting, we determined the longitudinal relaxation rate of solvent H_2O using an in-house inversion recovery experiment ($R_{1,H2O}$ = 0.175 s^{-1}) that contains an additional weak gradient to prevent water radiation dumping (see Methods). In general, to separate the MT pool of interest from the detected resonance (solvent water), we will refer to the dynamic properties of the MT pool via the T_2 relaxation constant (time-domain) while addressing the transverse relaxation properties of water by the R_2 relaxation rate (frequency domain), as R_2 rates and T_2 constants of a given molecule are inversely correlated and thus interchangeable. Furthermore, we estimated the chemical shift of the MT pool (agarose) via interpolation (5.3 ppm, main text, Fig. 3a). Global fitting of experimental data to our model yielded excellent agreement when assuming a Gaussian line shape for the agarose MT pool, with an overall residual deviation between our model and experimental data (fit deviation - see Methods) of 0.088 (main text, Extended Data Fig. 6a). In contrast, fitting this dataset assuming a super-Lorentzian line shape resulted in less agreement (fit deviation > 0.1, main text, Extended Data Fig. 6a). The requirement of a Gaussian line shape for the agarose, which consists of a rigid fiber network, agrees with theoretical considerations[3] and previous studies using agar, a structurally similar polysaccharide, as a model.[2] The rigidity of the agarose meshwork is further reflected by a T_2 relaxation constant of 17 µs, which aligns with a published T_2 value of 13 µs for agar.[2] Proton exchange between the agarose meshwork and bulk H_2O occurs at an exchange rate constant of 112 s^{-1}, while additional water molecules are physically associated with agarose, leading to an elevated transverse relaxation rate of bulk H_2O of 4.13 s^{-1}, compared to expected R_2 rates of 0.5-1 s^{-1} for water in the absence of an MT-pool [2,4,5] (main text, Fig. 3b). In our analyses, the MT-pool population directly reflects the concentration or, in case of RNA condensates, the amount of condensed RNA, as a fraction of protons relative to the overall

number of protons in our solutions. Thus, an agarose population of 0.07% can be interpreted as a percentage of total protons present in the solution belonging to the semi-solid agarose. On the other hand, the expected proton population of the agarose MT pool, based on a 0.5% (w/v) agarose solution, can be approximated using a molar concentration of 16 mM for agarose disaccharide units (D-galactose and 3,6-anhydro-L-galactose; MW ≈ 320 g/mol; [agarose] ≈ $\frac{5\,g/L}{320\,g/mol}$ ≈ 16 mM). Depending on the number of protons from these units which contribute to saturation transfer towards bulk H_2O, we anticipated a relative agarose proton population between 0.06% (considering the four hydroxyl protons: $\frac{4*16\,mM}{(2*53*10^3+4*16)\,mM} \cong 0.06\%$) and 0.25% (considering all 17 protons: $\frac{17*16\,mM}{(2*53*10^3+17*16)\,mM} \cong 0.25\%$). The relative MT-pool population of 0.07%, determined here, is therefore in reasonable agreement with our theoretical assumptions.

Supplementary Note 3: Testing for the required model complexity

Like any theoretical model used to describe biomolecular processes, the model employed for our CONDENSE-MT analyses is an approximation to the (5'-CAG-3')$_{31}$ RNA system we employed for our method development. Thus, the CONDENSE-MT model assumes one pool for agarose, which is quantified separately and held constant during quantification of RNA condensates, and one semi-solid pool for the RNA condensate itself. The latter implies that all RNA which is associated with the condensates can readily be described by one set of parameters which were optimized during global fitting. It is also important to consider more complex models, assuming the presence of different semi-solid RNA species, such as outer and inner layers of a given condensate. Fitting the effect of RNA condensates with one MT pool yielded reasonable results and very good agreement of the model with experimental data (main text, Fig. 4b, shown again for comparison in Supplementary Fig. 1a). Nevertheless, we repeated this analysis by adding one additional semi-solid RNA pool to the model and quantifying the physical parameters of both RNA pools simultaneously (at constant agarose parameters). This analysis, reassuringly, revealed that even the extended model yields almost identical parameters for one semi-solid RNA pool (Supplementary Fig. 1b, semi-solid pool 1). The second pool, however, is almost completely ignored during the computational optimization, as can be seen by a population of this pool of approximately 0% and otherwise strongly elevated parameter uncertainties (Supplementary Fig. 1b, semi-solid pool 2). We therefore conclude that the complexity of our CONDENSE-MT model (main text, Fig. 2b) is sufficient to fully describe the (5'-CAG-3')$_{31}$ model system which we used in this study. It may be noted, however, that application of CONDENSE-MT to more complex, multicomponent condensates might require adjustments of the model presented here towards more complex models.

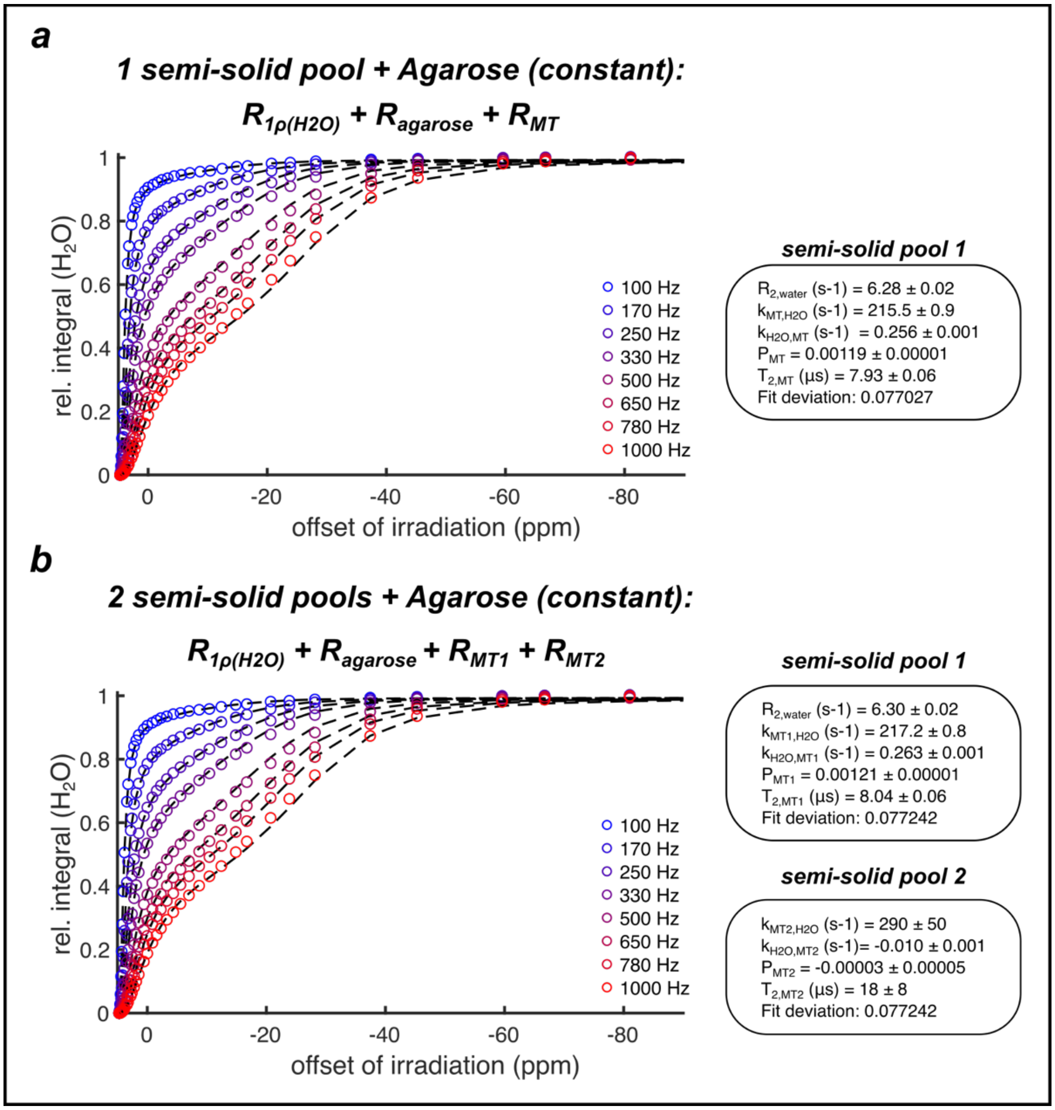

Supplementary Fig. 1: Effect of model complexity on CONDENSE-MT analyses

a Quantification of (5'-CAG-3')$_{31}$ condensates at 150 µM (5'-CAG-3')$_{31}$, embedded in agarose (0.5% w/v) via CONDENSE-MT. Physical parameters of agarose were previously determined and held constant during fitting. Experimentally recorded MT profile is shown as scatter, theoretical prediction after computational optimization is given as dashed line. Saturation amplitude (B_1) is given in Hz for each plot. Resulting physical parameters of condensed (5'-CAG-3')$_{31}$ are given to the right. Parameter errors were estimated from the diagonal elements of the variance-covariance matrix resulting from fitting experimental data to the CONDENSE-MT model. Fit deviation indicates overall deviation of experimental datapoints from the model prediction after computational optimization. **b** Quantification of (5'-CAG-3')$_{31}$ as shown in **a** with the assumption of two different semi-solid RNA pools for computational optimization. Computational fitting was initialized using the same starting parameters for optimization for both semi-solid pools. Physical parameters of agarose were previously determined and held constant during fitting. The parameters of both semi-solid pools were fitted simultaneously via 7-parameter global fitting. Resulting parameters for both semi-solid pools are given on the right.

Supplementary Note 4: Approximating tumbling rates of condensed RNA

The T_2 relaxation of condensed RNA is sensitive to conditions where we observed a pronounced reduction in droplet size (main text, Fig. 4c,d). Therefore, we considered whether CONDENSE-

MT reports on tumbling rates of the entire droplet instead of RNA motions within the condensate. For a viscous, semi-solid material, we would expect residual motions within the condensate at lower rotational correlation times than full-droplet tumbling. In contrast, RNA tumbling at rotational correlation times comparable to that of spherical droplets at 0.5-1 μm radius would point towards a more rigid, solid state of matter, suggesting the absence of residual internal motions. We employed the commonly known Debye-Stokes-Einstein theory for Brownian particle motion to approximate the rotational correlation time of entirely solid, spherical condensates, given by:[6]

$$\tau_R = \frac{4\pi\eta r^3}{3kT}$$

The viscosity of our agarose meshwork (0.5%, w/v) can be estimated to 0.004 Pa·s according to previous reports.[7] Bright field imaging indicated an average radius of 0.5-1 μm for (5'-CAG-3')$_{31}$ condensates at 55°C and 50 mM $MgCl_2$ (main text, Fig. 4d). From these approximations, we expect the droplets to tumble at 0.5-4 s rotational correlation time.
CONDENSE-MT reports on RNA tumbling, which results in transverse relaxation rates of nuclei in condensed RNA between 7 and 15 μs (main text, Fig. 4c, and Supplementary Fig. 2a). With knowledge of condensed RNA T_2 relaxation, we correlated transverse relaxation with molecular tumbling using the Bloembergen-Purcell-Pound (BPP) theory of two spins under dipole-dipole interaction,[8,9] via:

$$\frac{1}{T_2} = \frac{3}{20} \cdot b^2 \cdot (3J_0 + 5J_\omega + 2J_{2\omega}) = \frac{3}{20}\left(\frac{\mu_0 \hbar \gamma^2}{a^3}\right)^2 \left(3\tau_c + \frac{5\tau_c}{1+\omega^2\tau_c^2} + \frac{2\tau_c}{1+4\omega^2\tau_c^2}\right)$$

with the variable a as the average interproton distance in our system, τ_c as the rotational correlation coefficient of condensed RNA and μ_0 as the magnetic permeability through vacuum ($4\pi\cdot10^{-7}$ Tm/A). Under slow tumbling and at high magnetic field ($\omega^2\tau_c^2 >> 1$), we approximated the correlation between T_2 and τ_c by neglecting the Larmor frequency component (J_ω) and double-Larmor frequency component ($J_{2\omega}$), leading to:

$$\frac{1}{T_2} \approx \frac{b^2\tau_c}{10}$$

as a valid approximation for our system.

When assuming an interproton distance 1.5 Å for these approximations (Supplementary Fig. 2b, *blue line*), our calculations point to rotational correlation times in the low μs regime (5.6 μs rotational correlation coefficient at a T_2 relaxation constant of 7.8 μs, main text, Fig. 4 and

Supplementary Fig. 2a,b). As a control, increasing the estimated interproton distance through space in an RNA condensate to 3 Å led to an approximately 50-fold increase in the resulting rotational correlation time (359 μs, Supplementary Fig. 2b, *red line*). Therefore, for any reasonable assumption of the interproton distance for condensed RNA, the expected rotational tumbling rate is 5-6 orders of magnitude higher than expected for solid droplet tumbling (τ_c = 0.5-4 s, as described above). We therefore conclude that CONDENSE-MT reports on internal dynamics of condensed RNA in the dense phase, which occurs 3-4 orders of magnitude slower than free biomolecules in solution, as these typically tumble at nanosecond correlation times.

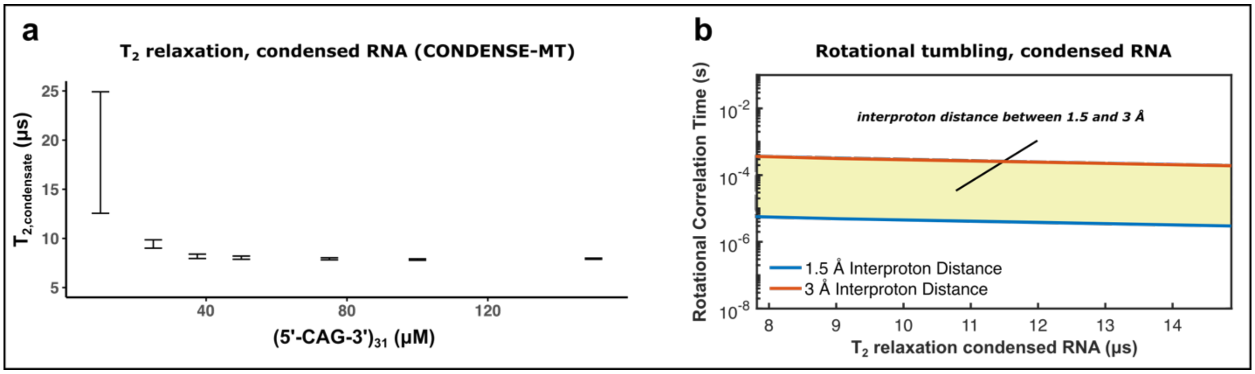

Supplementary Fig. 2: Rotational tumbling rates of condensed (5'-CAG-3')$_{31}$

a T_2 relaxation constants of biphasic (5'-CAG-3')$_{31}$ (55°C, 50 mM $MgCl_2$) as a function of overall RNA concentration. All data were obtained using CONDENSE-MT. Data are presented as error bars, centered to the respective best-fit value after computational optimization ± parameter uncertainties, which were estimated from the diagonal elements of the variance-covariance matrix resulting from fitting experimental data to the CONDENSE-MT model. **b** Approximated rotational correlation times for tumbling of a molecular species with T_2 relaxation constants between 7.8 and 15 μs according to NMR spin relaxation theory (Bloembergen-Purcell-Pound theory). The range of rotational correlation times of tumbling, corresponding to an average interproton distance between 1.5 and 3 Å, is indicated in yellow. Calculations were done assuming a proton Larmor frequency of 700 MHz and a magnetic permeability through vacuum (μ_0) of $4\pi \cdot 10^{-7}$ Tm/A.

Supplementary Note 5: Approximating condensed water populations in RNA condensates

CONDENSE-MT allows for detection and characterization of NMR-invisible, condensed RNA and is, in addition, sensitive to an increase in bulk water R_2 rates due to slow-tumbling, condensed water molecules (main text, Extended Data Fig. 5b and Supplementary Fig. 3a). Elevated bulk water relaxation rates in the presence of biomolecules, semi-solid-like molecular species, or condensates due to motion-restricted water were previously reported.[2,4,10] Accordingly, it was shown that bulk water relaxation can be described as a linear combination of free water relaxation and a contribution of fast relaxing, motion-restricted water which depends on the population of condensed or bound water molecules present in the overall system (Supplementary Fig. 3a). Due to the highly skewed populations between free and condensed water, we here assumed the population of free water to be 1. We therefore dissected the apparent relaxation rates of water into contributions of free water and condensed water relaxation as described previously:[10]

$$R_{2,app} = R_{2,free\ water} + P_{condensed\ water} * R_{2,condensed\ water}$$

$$R_{1,app} = R_{1,free\ water} + P_{condensed\ water} * R_{1,condensed\ water}$$

Furthermore, the established framework of NMR spin relaxation of two spins under through-space dipolar interactions (Bloembergen-Purcell-Pound theory) allows for a general expression of relaxation rates as a function of molecular tumbling via the rotational correlation coefficient τ_c (simulations of the relationship between tumbling and relaxation rates according to these formalisms are shown in Supplementary Fig. 3b):[8–10]

$$R_2 = \frac{3}{20}\left(\frac{\mu_0 \hbar \gamma^2}{a^3}\right)^2 \left(3\tau_c + \frac{5\tau_c}{1+\omega^2\tau_c^2} + \frac{2\tau_c}{1+4\omega^2\tau_c^2}\right)$$

$$R_1 = \frac{3}{10}\left(\frac{\mu_0 \hbar \gamma^2}{a^3}\right)^2 \left(\frac{\tau_c}{1+\omega^2\tau_c^2} + \frac{4\tau_c}{1+4\omega^2\tau_c^2}\right)$$

In our approach, we obtained information about $R_{1,app}$ from inversion recovery experiments (Supplementary Note 1 and main text, Methods), while CONDENSE-MT provided knowledge of $R_{2,app}$. As we aimed for a quantification for the relative increase in apparent water relaxation as a function of RNA condensate population, we approximated the R_1 and R_2 rates of free water as the water relaxation properties in absence of RNA (but in presence of the agarose meshwork). This assumption is valid as we next extracted the population of condensed water relative to the population of condensed RNA by global fitting of the linearly increasing water R_1 and R_2 rates as a function of the population of condensed (5'-CAG-3')$_{31}$ (main text, Fig. 4f and Extended Data Fig. 9c). By this approach, we translated the condensate-mediated increase in solvent relaxation into the relative population of condensed water per unit of condensed RNA, which we interpret as

the excess of water-protons per RNA proton in the dense phase. We obtained a rotational correlation coefficient of 3.6 ns and a relative population of 26 water protons per RNA proton in (5'-CAG-3')$_{31}$ condensates (main text, Extended Data Fig. 9c). Thus, water sequestered into the RNA condensates tumbles at rotational correlation coefficients similar to kDa-sized proteins in solution and three orders of magnitude slower than free water in solution. Importantly, for these calculations, we excluded datasets reporting on incomplete condensation and therefore only considered data where the concentration of (5'-CAG-3')$_{31}$ was 37.5 μM or higher (main text, Fig. 4c and Extended Data Fig. 9c).

According to our CONDENSE-MT results on RNA at different lengths, the T_2 relaxation of condensed RNA and the partition coefficient of RNA upon condensation were not affected by variations in CAG repeat number (main text, Fig. 5a,b and Extended Data Fig. 9a,b). We therefore assumed a fixed tumbling rate of 3.6 ns (and thus constant relaxation properties) of condensed water for all CAG condensates at different RNA lengths. This assumption enabled us to approximate the relative population of condensed water for (5'-CAG-3')$_{10}$ - (5'-CAG-3')$_{44}$ across several molar concentrations of total RNA for each RNA oligomer by directly converting apparent water relaxation rates into populations of condensed water for each dataset (Supplementary Fig. 3c). Here, we excluded all datasets where we detected condensed RNA at a population below 0.025% as we expect our assumption of fixed condensed water relaxation to be not valid for these datasets due to incomplete condensation and elevated rotational tumbling rates of condensed RNA (see main text Fig. 5b). Finally, given that the relative water content of condensed (5'-CAG-3')$_{31}$ does not depend on overall RNA concentration (main text, Fig. 4f), we assumed that the relative water content of CAG RNA of different length is similarly independent of overall RNA concentration (under conditions of full condensation). We therefore estimated the concentration-independent condensate water content for each CAG oligomer (main text, Fig. 5f) as the average water content for a given RNA length using previously determined hydration states for the respective oligomers at different concentrations (Supplementary Fig. 3c and main text, Fig. 5e).

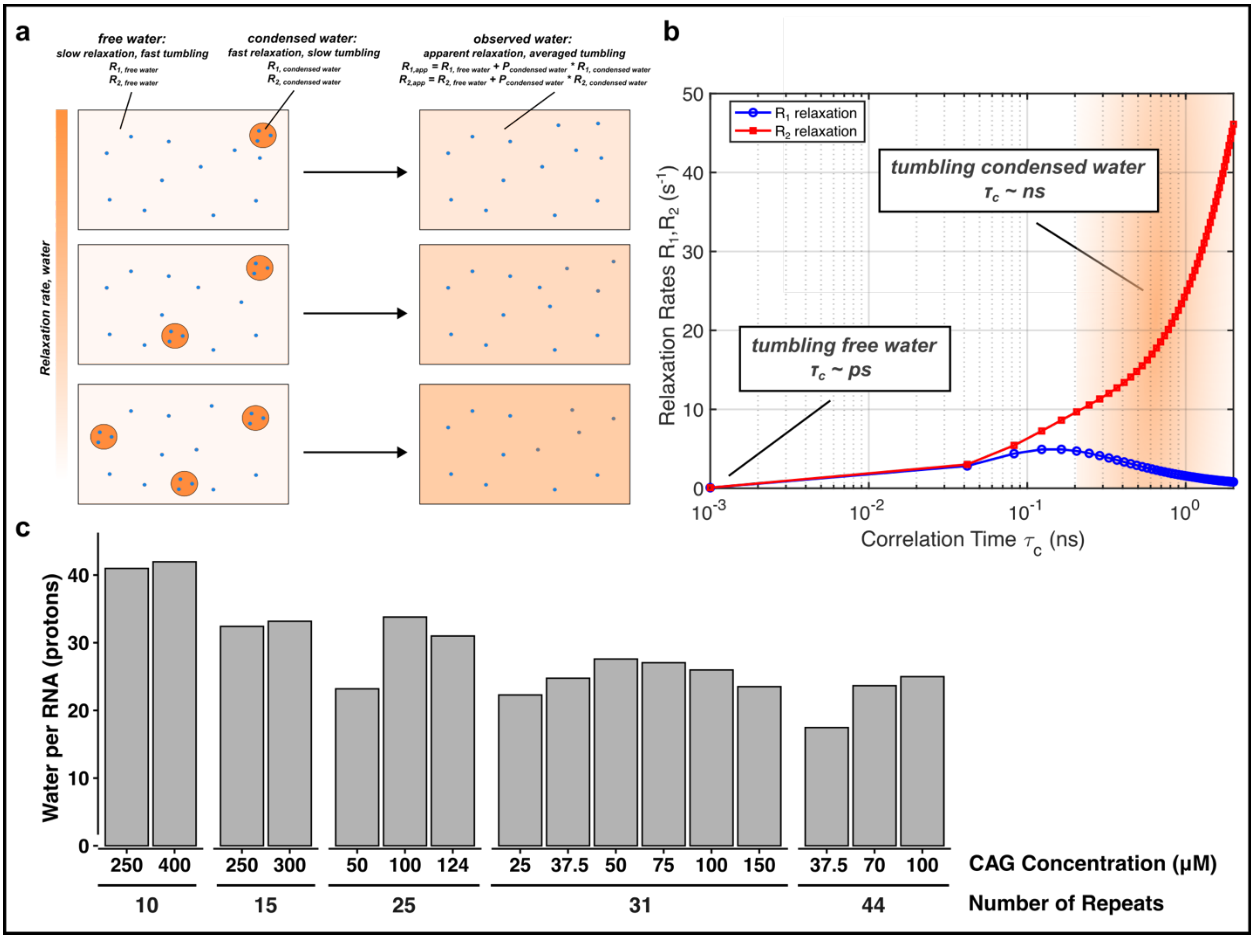

Supplementary Fig. 3: Approximating condensed water in RNA condensates

a Schematic representation of observed water relaxation properties, depending on the amount of a slow-tumbling, condensed water present in the system. Condensed water is given as circled areas, the overall amount of water in the system is represented by the overall box area. Relaxation rates for each water pool are indicated by color code, as shown on the left. **b** Simulated relaxation rates as a function of the rotational correlation coefficient of a nucleus under the assumption that trough-space dipolar interactions are the main relaxation source (Bloembergen-Purcell-Pound theory). The simulations were performed assuming a proton Larmor frequency of 700 MHz, a magnetic permeability through vacuum (μ_0) of $4\pi \cdot 10^{-7}$ Tm/A and an average, interproton distance of 1.5 Å. **c** Calculated proton ratio of water to RNA for RNA condensates as a function of RNA length at different molar concentrations of each RNA oligomer. Populations of water were determined assuming a rotational tumbling coefficient of 3.6 ns for condensed water, using overall water R_2 rates obtained via CONDENSE-MT and water R_1 rates determined via inversion recovery. Each bar plot resembles one NMR dataset (N = 1).

References

1. Morrison, C. & Mark Henkelman, R. A Model for Magnetization Transfer in Tissues. *Magn Reson Med* **33**, 475–482 (1995).
2. Henkelman, R. M. *et al.* Quantitative interpretation of magnetization transfer. *Magn Reson Med* **29**, 759–766 (1993).
3. Morrison, C., Stanisz, G. & Henkelman, R. M. Modeling magnetization transfer for biological-like systems using a semi-solid pool with a super-Lorentzian lineshape and dipolar reservoir. *J Magn Reson B* **108**, 103–113 (1995).
4. Karjalainen, J., Henschel, H., Nissi, M. J., Nieminen, M. T. & Hanni, M. Dipolar Relaxation of Water Protons in the Vicinity of a Collagen-like Peptide. *J. Phys. Chem. B* **2022**, (2538).
5. Hazlewood, C. F., Chang, D. C., Nichols, B. L. & Woessner, D. E. Nuclear Magnetic Resonance Transverse Relaxation Times of Water Protons in Skeletal Muscle. *Biophys J* **14**, 583–606 (1974).
6. Costigliola, L., Heyes, D. M., Schrøder, T. B. & Dyre, J. C. Revisiting the Stokes-Einstein relation without a hydrodynamic diameter. *Journal of Chemical Physics* **150**, (2019).
7. Ghebremedhin, M., Seiffert, S. & Vilgis, T. A. Physics of agarose fluid gels: Rheological properties and microstructure. *Curr Res Food Sci* **4**, 436–448 (2021).
8. Bloembergen, N., Purcell, E. M. & Pound, R. V. Relaxation Effects in Nuclear Magnetic Resonance Absorption. *Physical Review* **73**, 679–712 (1948).
9. Solomon, I. Relaxation Processes in a System of Two Spins. *Physical Review* **99**, 559–565 (1955).
10. K.-DASZKIEWICZ, O., HENNEL, J. W., LUBAS, B. & SZCZEPKOWSKI, T. W. Proton Magnetic Relaxation and Protein Hydration. *Nature* **200**, 1006–1007 (1963).
