# Supplementary material for: Water-detected NMR allows dynamic observations of repeat-expansion RNA condensates
Source: Nat Chem. 2025 Oct 15;17(11):1785–94. doi: 10.1038/s41557-025-01968-9 (PMC12580330; doi:10.1038/s41557-025-01968-9)

Extended Data Fig. 1b

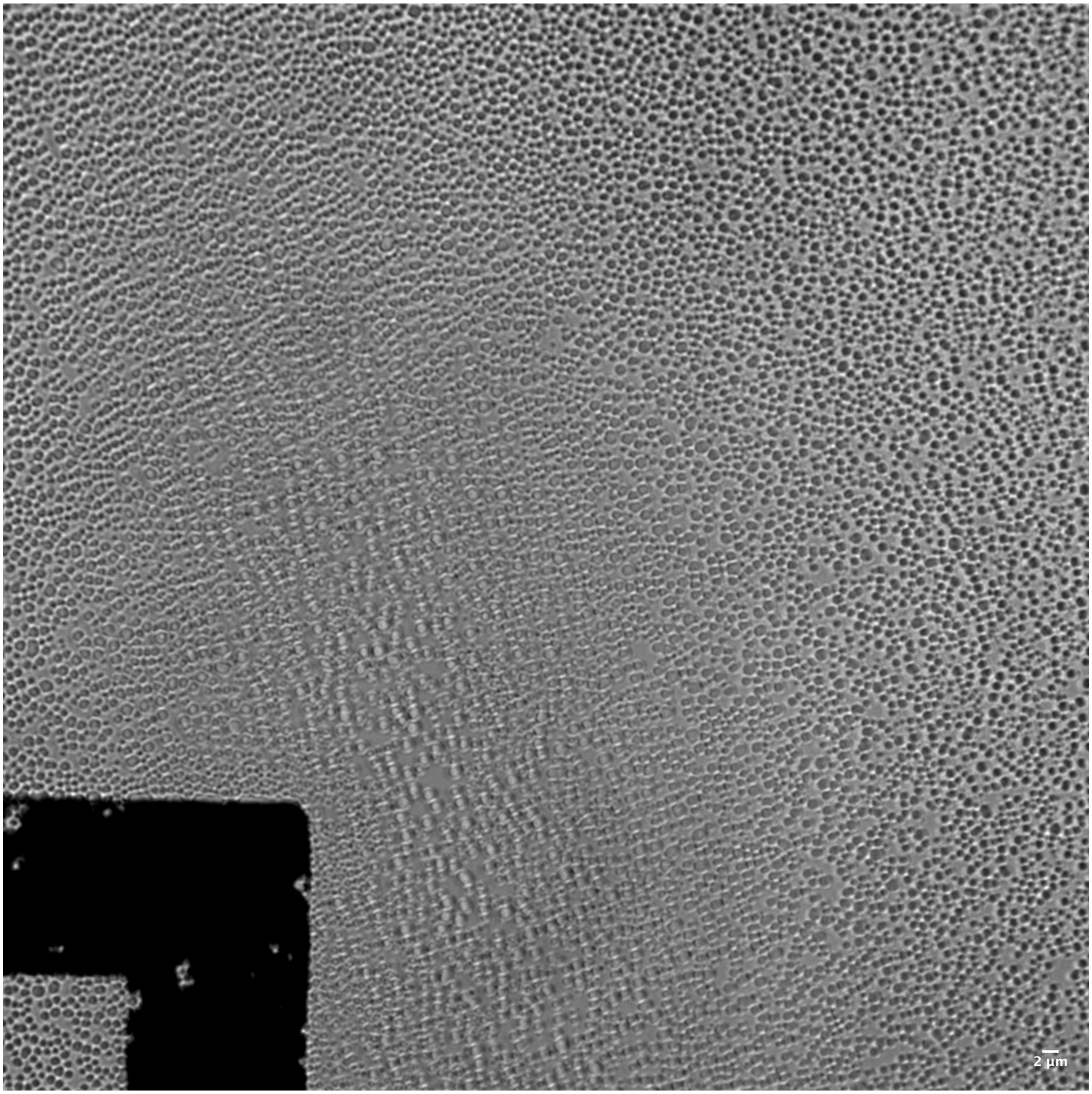

Extended Data Fig. 1c

Condensed (5’-CAG-3’)$_{31}$ NMR sample, with agarose

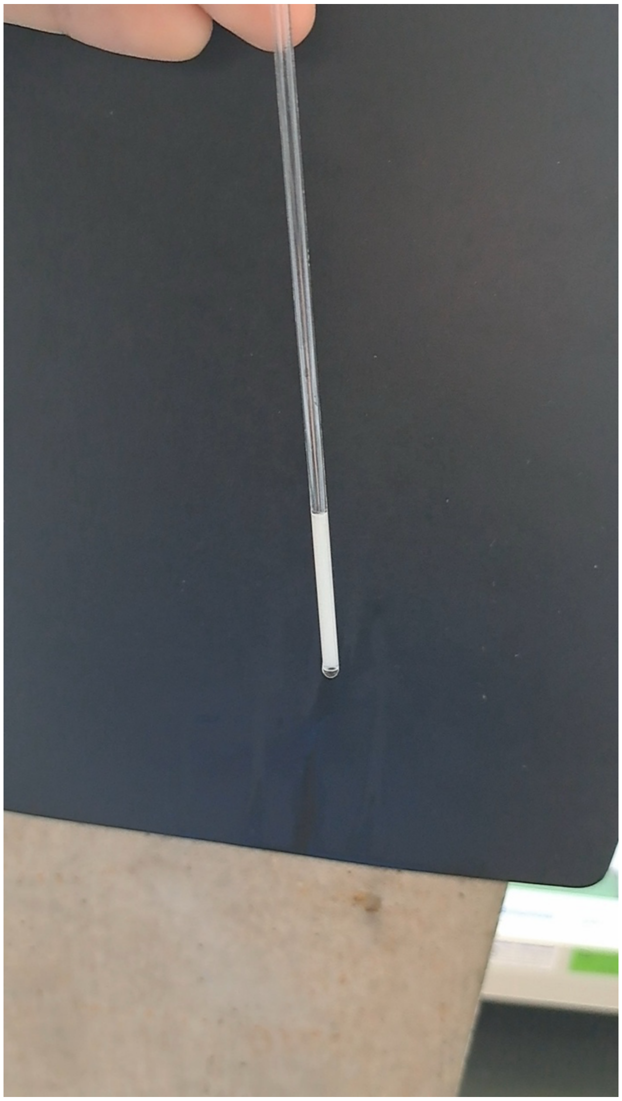

Condensed (5’-CAG-3’)$_{31}$ NMR sample, without agarose

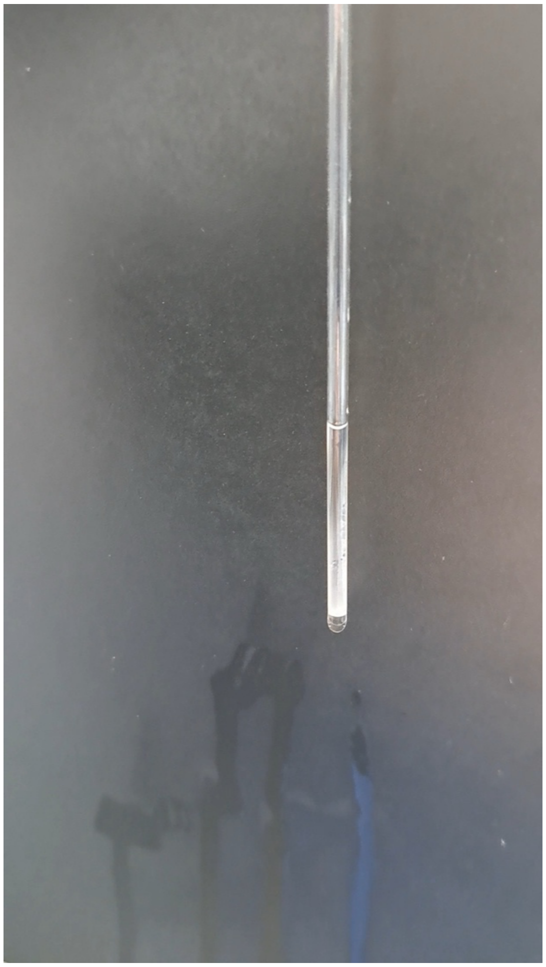

Supplement: Supplementary file 3 — Numerical source data for Figs. 1–5 and Extended Data Figs. 1, 4 and 6–9 and Table 1; uncropped images for Fig. 4 and Extended Data Fig. 1. [file 41557_2025_1968_MOESM3_ESM.zip › Source_Data_ED_Fig_1_images.pdf]
