# Supplementary material for: Water-detected NMR allows dynamic observations of repeat-expansion RNA condensates
Source: Nat Chem. 2025 Oct 15;17(11):1785–94. doi: 10.1038/s41557-025-01968-9 (PMC12580330; doi:10.1038/s41557-025-01968-9)

(5’-CAG-3’)$_{31}$, 150 μM

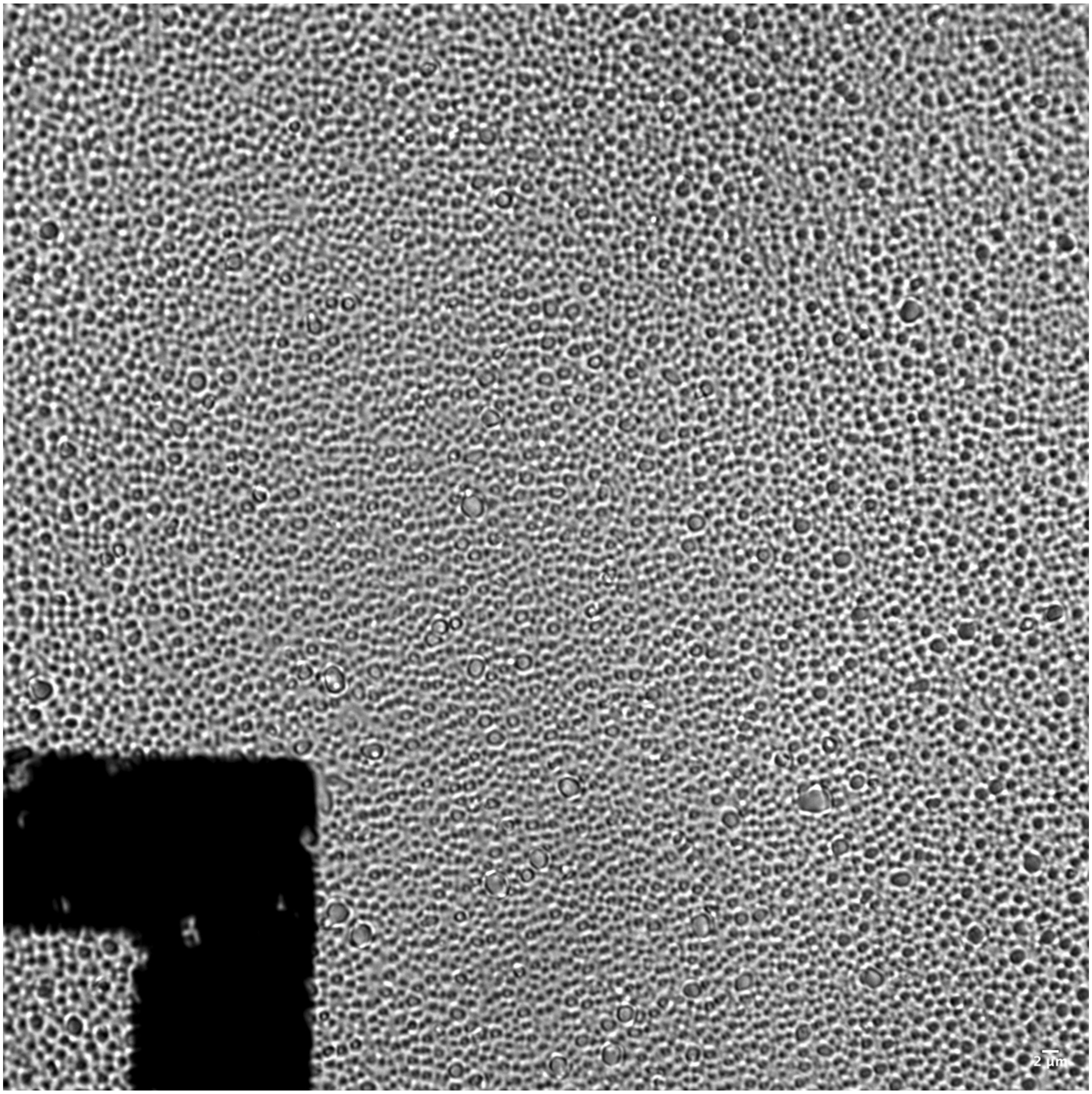

(5’-CAG-3’)$_{31}$, 100 μM

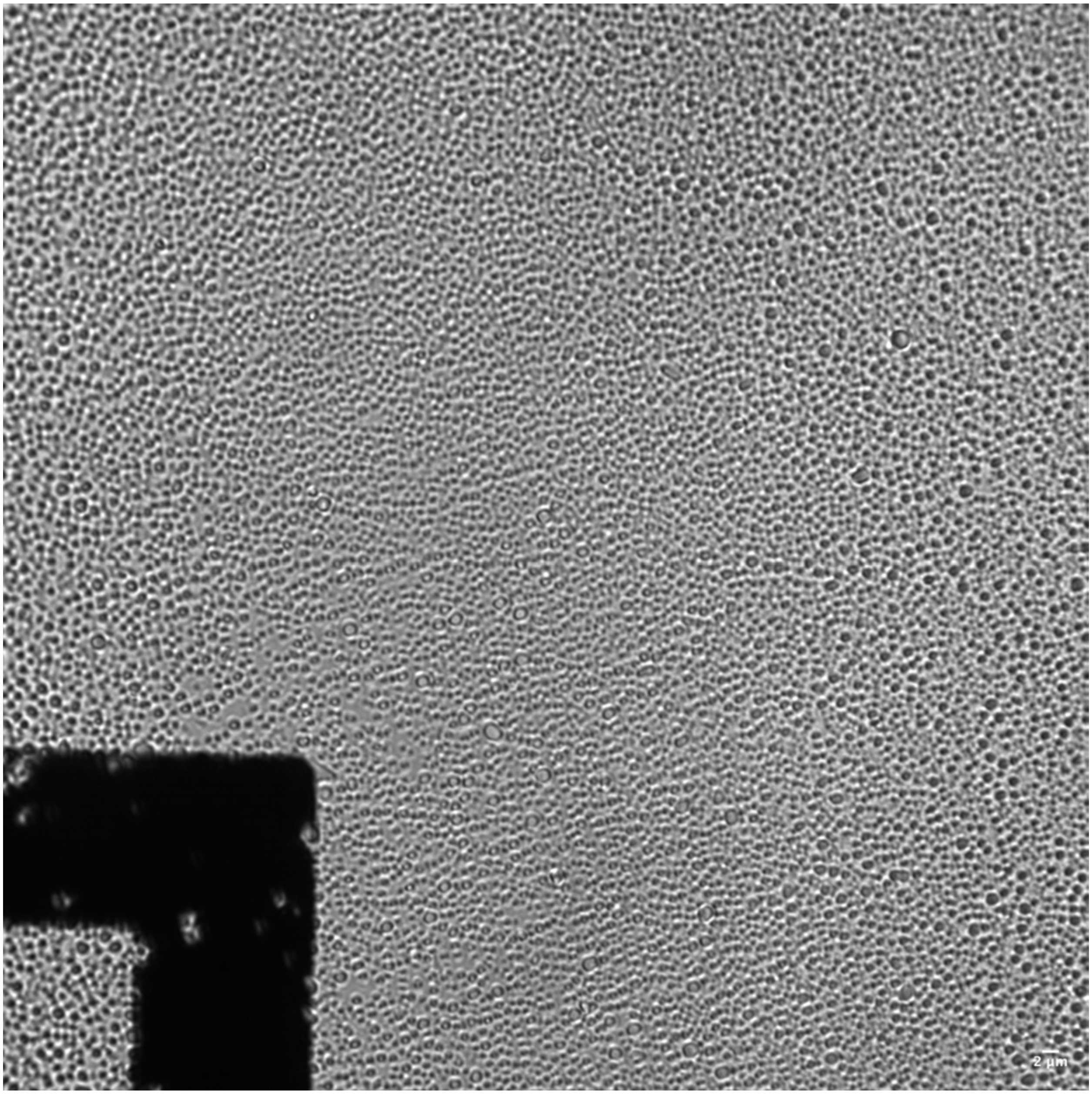

(5’-CAG-3’)$_{31}$, 75 μM

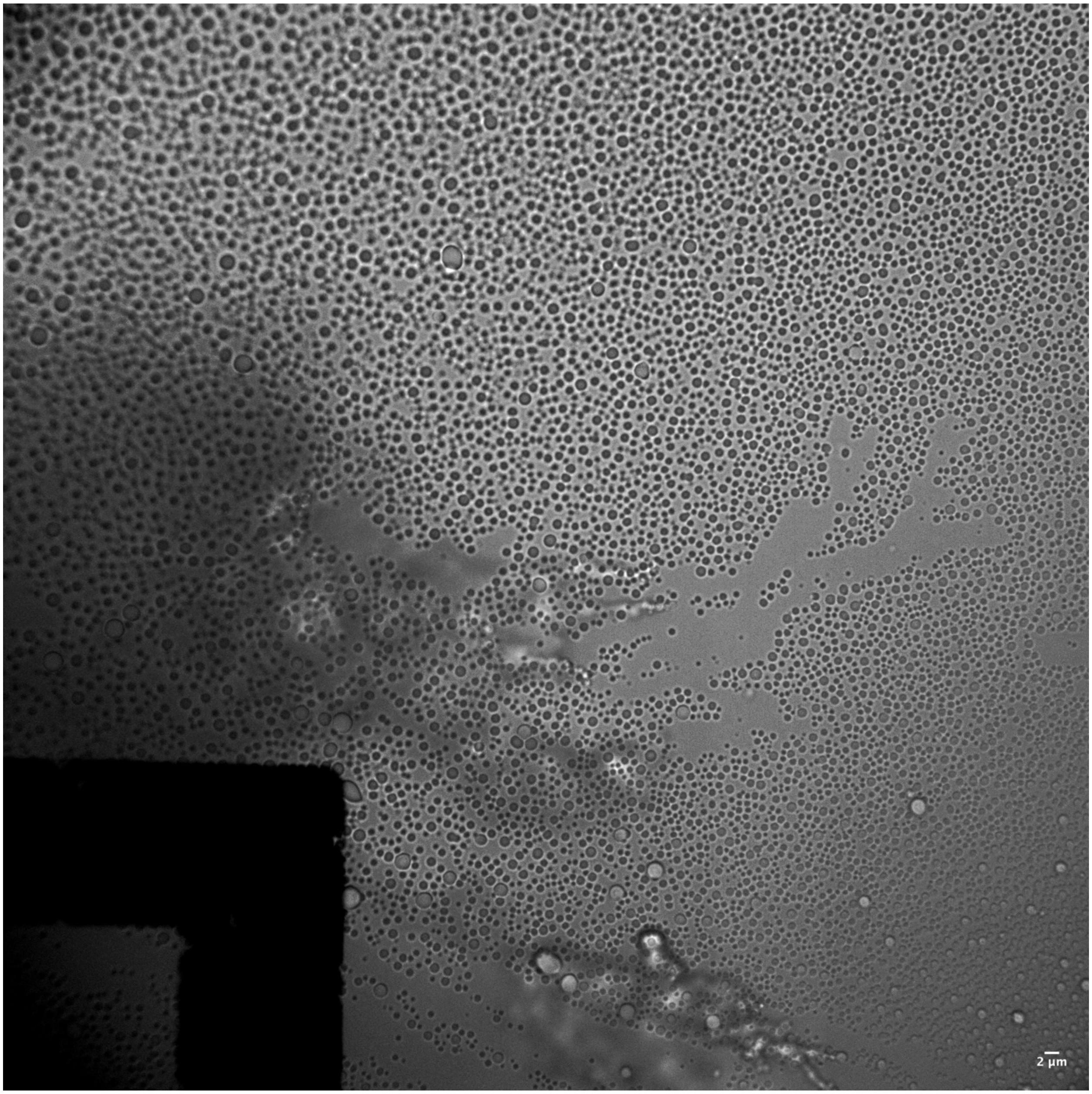

(5'-CAG-3')$_{31}$, 50 μM

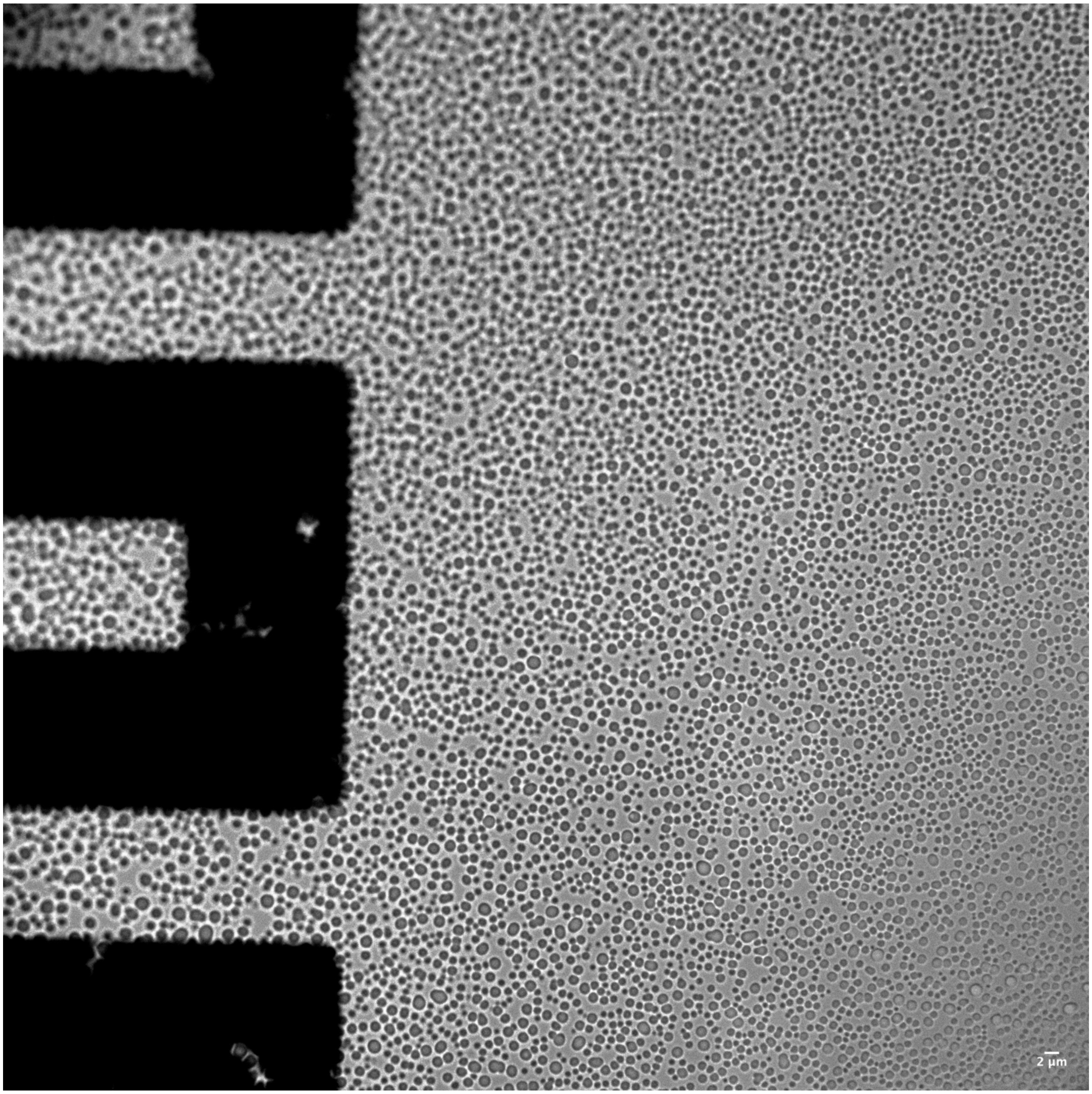

$(5'\text{-CAG-3'})_{31}$, 37.5 μM

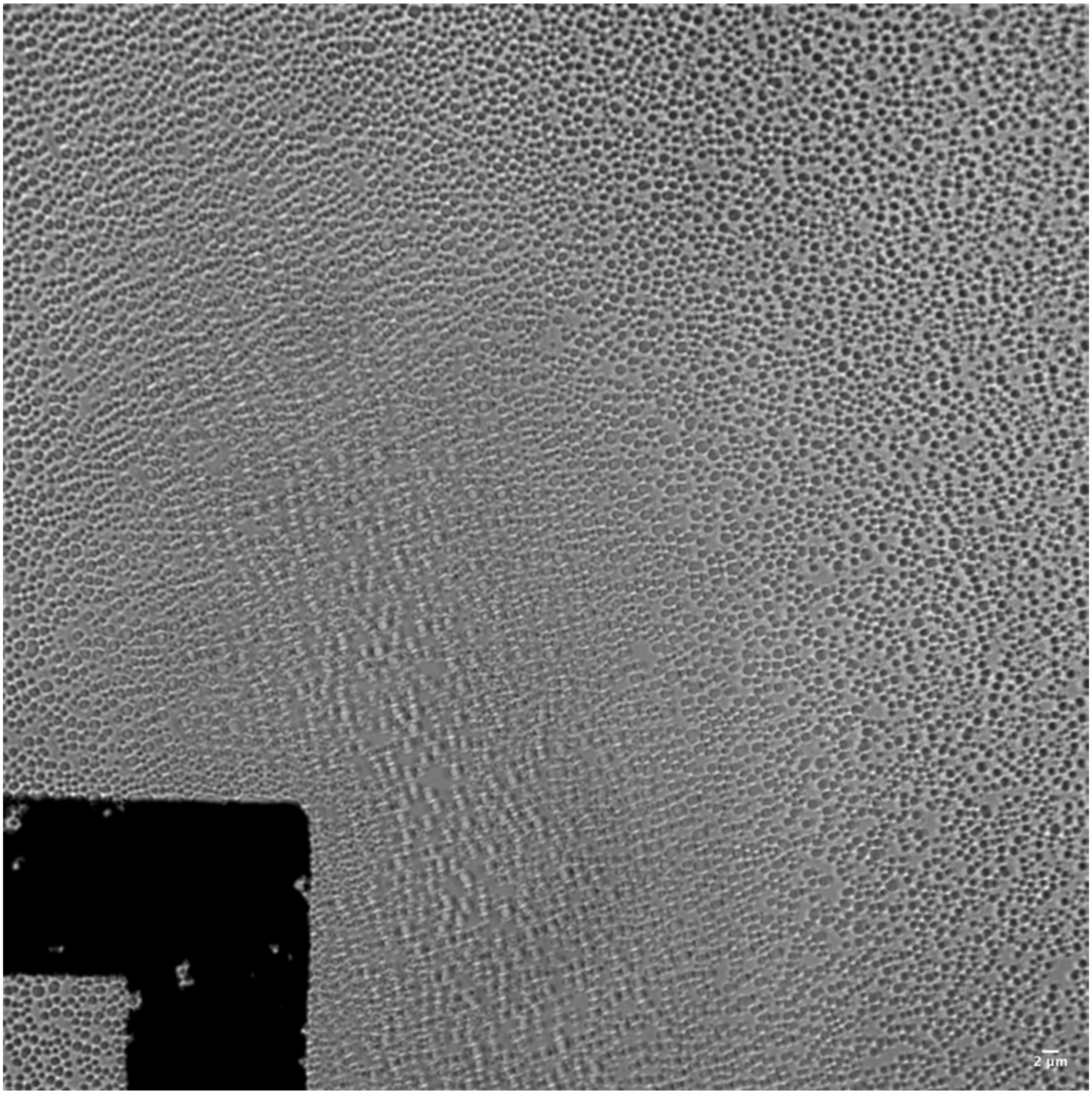

$(5'-CAG-3')_{31}$, 25 μM

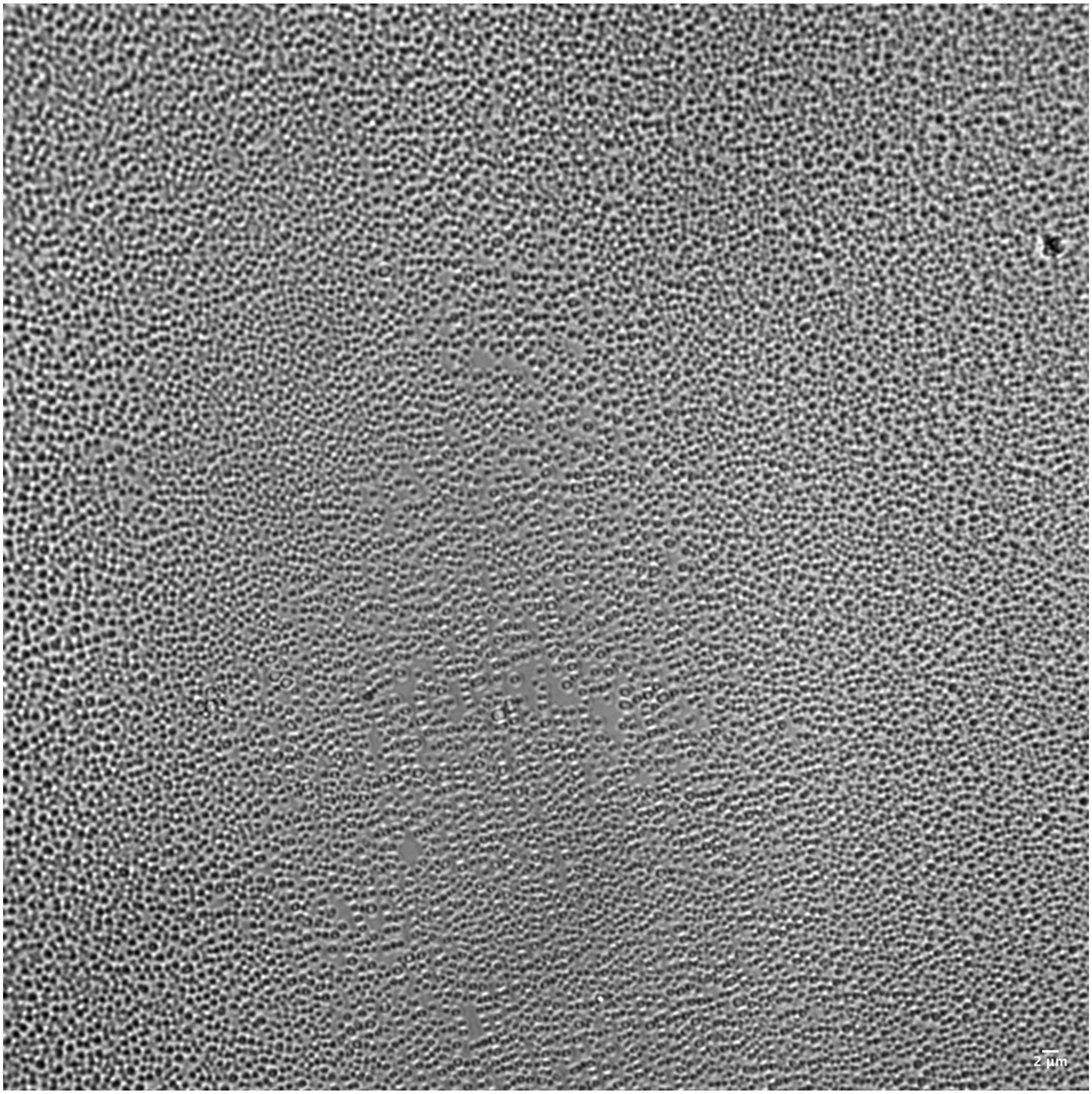

(5’-CAG-3’)$_{31}$, 10 μM

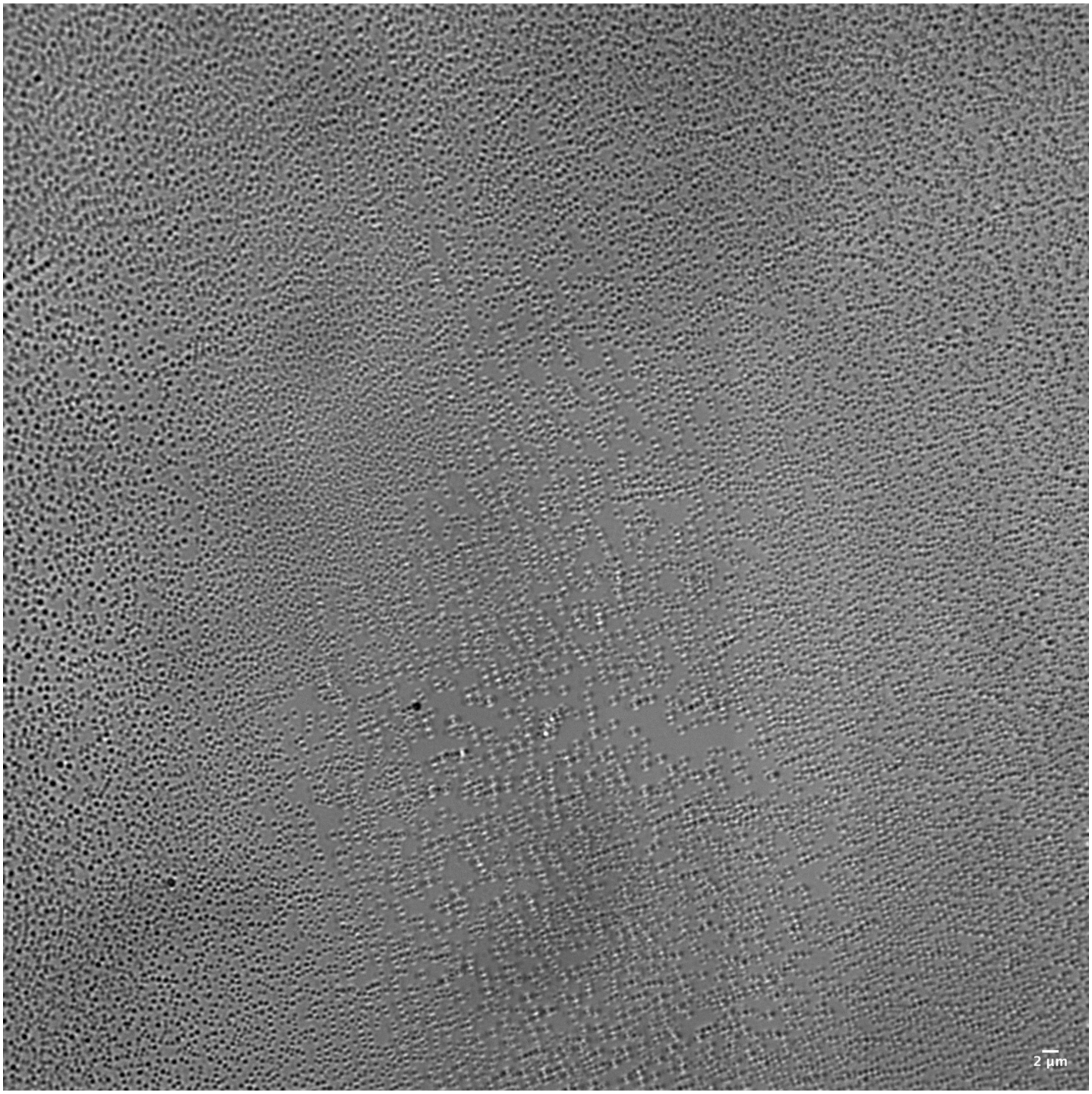

Supplement: Supplementary file 3 — Numerical source data for Figs. 1–5 and Extended Data Figs. 1, 4 and 6–9 and Table 1; uncropped images for Fig. 4 and Extended Data Fig. 1. [file 41557_2025_1968_MOESM3_ESM.zip › Source_Data_Fig_4_images.pdf]
